# SNARE-Mediated Exocytosis in Neuronal Development

**DOI:** 10.3389/fnmol.2020.00133

**Published:** 2020-08-07

**Authors:** Fabio L. Urbina, Stephanie L. Gupton

**Affiliations:** ^1^Department of Cell Biology and Physiology, University of North Carolina at Chapel Hill, Chapel Hill, NC, United States; ^2^UNC Neuroscience Center, Chapel Hill, NC, United States; ^3^UNC Lineberger Comprehensive Cancer Center, Chapel Hill, NC, United States; ^4^Carolina Institute for Developmental Disabilities, University of North Carolina at Chapel Hill, Chapel Hill, NC, United States

**Keywords:** vesicle, fusion pore, full vesicle fusion, kiss and run, neuronal development

## Abstract

The formation of the nervous system involves establishing complex networks of synaptic connections between proper partners. This developmental undertaking requires the rapid expansion of the plasma membrane surface area as neurons grow and polarize, extending axons through the extracellular environment. Critical to the expansion of the plasma membrane and addition of plasma membrane material is exocytic vesicle fusion, a regulated mechanism driven by soluble N-ethylmaleimide-sensitive factor attachment proteins receptors (SNAREs). Since their discovery, SNAREs have been implicated in several critical neuronal functions involving exocytic fusion in addition to synaptic transmission, including neurite initiation and outgrowth, axon specification, axon extension, and synaptogenesis. Decades of research have uncovered a rich variety of SNARE expression and function. The basis of SNARE-mediated fusion, the opening of a fusion pore, remains an enigmatic event, despite an incredible amount of research, as fusion is not only heterogeneous but also spatially small and temporally fast. Multiple modes of exocytosis have been proposed, with full-vesicle fusion (FFV) and kiss-and-run (KNR) being the best described. Whereas most *in vitro* work has reconstituted fusion using VAMP-2, SNAP-25, and syntaxin-1; there is much to learn regarding the behaviors of distinct SNARE complexes. In the past few years, robust heterogeneity in the kinetics and fate of the fusion pore that varies by cell type have been uncovered, suggesting a paradigm shift in how the modes of exocytosis are viewed is warranted. Here, we explore both classic and recent work uncovering the variety of SNAREs and their importance in the development of neurons, as well as historical and newly proposed modes of exocytosis, their regulation, and proteins involved in the regulation of fusion kinetics.

## The Remarkable Mammalian Neuron

### Development of Neurons

The human brain is one of the most complex structures in nature, containing an astounding ~8^11^ neurons and ~8^11^ non-neuronal cells and 12^13^–12^14^ synaptic connections in a roughly 1,400 cm^3^ volume (Drachman, 2005; Azevedo et al., 2009; Micheva et al., 2010). The establishment of precise and complex neural networks in the human brain is a major developmental task; this continues to be an area of rich scientific investigation. Neurons are specialized cells, with both a uniquely polarized structure and polarized function. The extremely polarized morphology of neurons is typified by a single axon and multiple dendrites. During development, the axon extends outward from the neuronal cell body, also known as the soma, toward future synaptic partners. Eventually, synapses form between the axon and these synaptic partners. Dendrites, which receive synaptic signals, are similarly often highly ramified structures that receive input from multiple axons. Such axonal-dendritic synaptic connections constitute the neural networks that orchestrate complex behaviors, such as those of the mammalian brain. Many outstanding questions regarding neuronal development remain incompletely answered, including how do neurons sense their extracellular environment and use this information to expand in both volume and surface area, and consequently create stereotypical network connections. In this review, we explore the role that proteins of the soluble N-ethylmaleimide-sensitive factor attachment proteins receptor (SNARE) family and SNARE-mediated exocytosis play in plasma membrane expansion and synaptic function in different stages of neuron development, outgrowth, and function. We highlight recent improvements in technology that have allowed a closer examination of SNARE complex composition, regulation of the fusion pore, and different modes of exocytosis, reinvigorating old questions and proposing new ones in neuronal biology.

**Figure 1:**
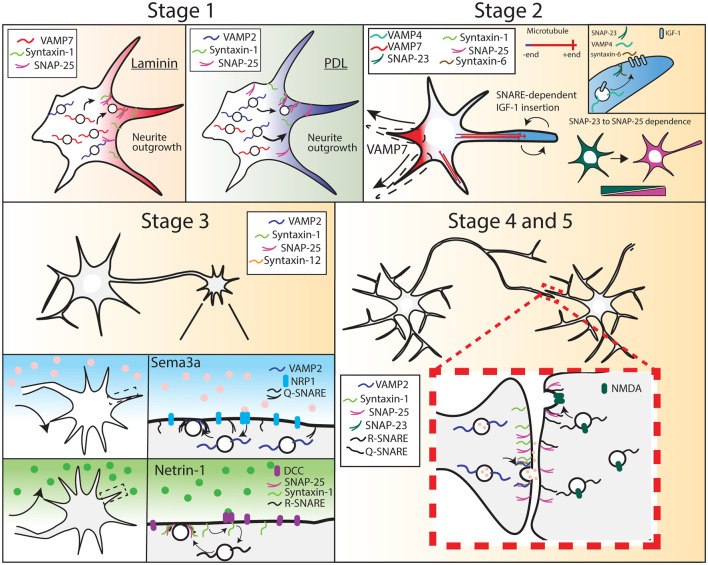
Soluble N-ethylmaleimide-sensitive factorattachment proteins receptors (SNARE) expression and function during different stages of neuron development. Stage 1, R-SNARE usage is context-dependent, as cortical neurons grown on laminin require VAMP7 for neurite outgrowth, whereas neurons grown on PDL require VAMP2. Stage 2, a multitude of SNAREs are required for neurite outgrowth and axon specification. Neurite outgrowth becomes VAMP7-dependent, and soluble NSF-attachment proteins (SNAP-25) expression increases while SNAP-23 expression decreases. Stage 3, VAMP2, SNAP-25, and syntaxin-1 are required for axon attraction and repulsion in response to chemotrophic factors. Stage 4 and 5, VAMP2, syntaxin-1, and SNAP-25 are required at the pre-synapse for synaptic transmission. SNAP-25 is required at the post-synapse for SNARE-mediated NMDA receptor insertion.

Neuronal development is characterized by a progression of morphological events. After birth from neural progenitor cells, immature neurons migrate to specified locations before neuritogenesis (Cooper, 2013). For example, glutamatergic neurons born in the ventricle wall form a bipolar morphology and migrate to specified layers of the cortex, dependent upon their birthdate, with early-born neurons forming deeper layers of the cortex and later-born neurons comprising more superficial cortical layers. Upon reaching their destination, neurons progress through several stereotypical morphological stages during development, which is recapitulated with *in vitro* neuronal culture (Dotti et al., 1988; Bradke and Dotti, 1997). This progression begins when multiple immature neurites extend from the soma, in a process termed neuritogenesis (Figure 1, Stage 1). Subsequently, one neurite is specified as the axon through several molecular events, including the accumulation of axonal components, whereas the remaining neurites accumulate dendritic components (Wisco et al., 2003; Kaibuchi, 2009). Axon specification leads to differences in polarized intracellular transport of materials into these two regions of the neuron (Figure 1, Stage 2; Sampo et al., 2003; Wisco et al., 2003). Microtubules are polarized anterogradely in the axon, with growing plus ends unidirectionally facing away from the cell body. In contrast, dendrites exhibit bipolar microtubule orientation, with approximately equal numbers of microtubule plus-ends facing anterograde and retrograde (Baas et al., 1988; Stepanova et al., 2003). How this distinct microtubule polarity intrinsically alters the delivery and sites of fusion for exocytic cargo remains an outstanding question.

Following axon specification, the axon matures *via* several additional stages of growth. The growing axon extends toward targets of innervation (Figure 1, Stage 3). This directional growth is led by the growth cone, a sensory and guidance machine, which navigates in response to extracellular guidance cues, trophic factors, and extracellular matrix components (Gomez and Zheng, 2006; Tojima et al., 2011; Tojima, 2012; Dudanova and Klein, 2013; Sutherland et al., 2014; Akiyama and Kamiguchi, 2015). Axon branching, another stage in neuronal morphogenesis, increases axonal territory and size, allowing the axon to ultimately form synapses with multiple partners (Figure 1, Stage 4, and 5; Bilimoria and Bonni, 2013). These connections are subsequently strengthened, weakened, and pruned over the lifespan of an organism, through processes of synaptic plasticity. The proper connectivity, maintenance, and plasticity of synapses are critical for appropriate physiological and behavioral actions, disruption of which can lead to disease (Lüscher and Isaac, 2009; Schulz and Hausmann, 2016).

The developmental extension and branching of axon and dendrites involve a significant expansion of neuronal surface area (Pfenninger and Friedman, 1993). Indeed, the expansive plasma membrane surface area of a neuron is considerably larger for example than a simpler-shaped cell. For example, a mammalian neuron has an average surface area of ~250,000 μm^2^ up to millions of square microns, whereas a fibroblast has an average surface area orders of magnitude smaller, at approximately 3,600 μm^2^ (Steinman et al., 1983; Pfenninger and Friedman, 1993). This large neuronal surface area highlights the critical importance of the insertion of membrane material during neuronal development. The primary driver of neuronal surface area expansion, at least early in neuronal development, is thought to be exocytosis, a fundamental cellular mechanism in which a vesicle fuses with the plasma membrane, forming one contiguous surface.

Two distinct vesicle populations exist in mature neurons: small synaptic vesicles, which generally contain low–molecular weight neurotransmitters, and dense-core vesicles, in which neuropeptides and neurotrophins are packaged. Synaptic vesicles are smaller, typically averaging 40 nm in diameter, whereas dense-core vesicles average ~100 nm in diameter. Therefore these vesicles contribute different amounts of surface area material upon fusion (Qu et al., 2009; Merighi, 2018). Additionally, the regulation of their fusion is likely distinct. Dense-core vesicle fusion requires repetitive and prolonged stimulation compared to synaptic vesicle fusion (Lundberg et al., 1986; Hartmann et al., 2001; Balkowiec and Katz, 2002; Frischknecht et al., 2008). The distinct sensitivity of these vesicle populations suggests different fusion machinery or protein regulation between the two. Even though these different vesicle types are known to exist in mature neurons, their relative contributions to membrane expansion in developing neurons are not known. Further, both vesicle types are below the diffraction limit of light, and thus there are unknown differences in fusion parameters between the two. As such, this review frequently does not differentiate between vesicle types.

Exocytosis is mediated by Soluble-NSF-attachment protein receptors (SNAREs) proteins, which form a stable complex thought to provide the energy needed for the fusion of opposing lipid bilayers (Südhof and Rothman, 2009; Jahn and Fasshauer, 2012). Additional mechanisms that add plasma membrane exist, such as lipid transfer at endoplasmic reticulum-plasma membrane contact sites (Yu et al., 2016; Nath et al., 2019). Exogenous membrane addition can also occur later in development *via* lipoprotein particles donated by glial cells (Frühbeis et al., 2013; Lewis, 2013). The relative contribution of direct lipid transfer and glial donation of plasma membrane material remains relatively unexplored in literature; exocytosis is thought to provide sufficient membrane for early stages of neuronal development (Urbina et al., 2018, 2020). SNARE-mediated membrane growth plays an essential role in neuronal development, mediating neurite outgrowth, neuron polarization, growth cone guidance, synapse formation, and ultimately functional synaptic transmission.

### SNAREs and Exocytosis

#### Discovery of the Fusion Machinery

The search for the proteins involved in transport between membrane-bound compartments in eukaryotic cells identified several components of the fusion machinery. The Rothman lab discovered and named N-ethylmaleimide-sensitive factor (NSF), a cytosolic ATPase required for fusion reactions between membranes, from mammalian cells (Block et al., 1988). Using NSF as bait, they identified soluble NSF-attachment proteins (SNAPs), *via* their formation of NSF-SNAP complexes (Malhotra, 1988; Clary et al., 1990; Rothman and Orci, 1992). Mutations in genes encoding homologs in yeast for NSF and SNAPs cause secretory defects (Crary and Haldar, 1992; Novick and Schekman, 1979; Shah and Klausner, 1993). A search for SNAP REceptors (SNAREs) using bovine brain identified synaptobrevin-2 (VAMP-2), SNAP-25 (coincidentally named SNAP, unrelated to the SNAPs of NSF), and syntaxin-1(Söllner et al., 1993) and revealed that these three SNAREs formed a complex. VAMP-2, SNAP-25, and syntaxin-1 were sufficient to fuse membranes in liposomal and cell-cell fusion assays, and thus the minimal machinery necessary for fusion, conserved in eukaryotes, had been discovered (Weber et al., 1998; Hu et al., 2003).

#### SNAREs in Complex

The crystal structure of the SNARE complex and the identification of a 60–70 amino acid sequence conserved in all SNARE proteins, the SNARE motif, suggested a molecular mechanism for fusion (Sutton et al., 1998). SNAREs attached to separate membranes zipper into a tight complex, bringing opposing membranes into contact and providing sufficient energy to open a fusion pore (Gao et al., 2012). Four SNARE motifs (two from SNAP-25, one from syntaxin-1, and one from VAMP-2) are oriented into a parallel four α-helical bundle composed of leucine, isoleucine, and valine residues. The SNARE-complex bundle is thus formed of stacked leucine zipper-like layers. Within the leucine-zipper layers, there is a highly conserved and completely buried “0” layer composed of ionic interactions between an arginine (R) from VAMP-2, glutamine (Q) from syntaxin-1, and two Q from SNAP-25. The flanking leucine-zipper layers act as a water-tight seal to shield the ionic interactions of the R and three Q from the surrounding solvent. This seal further stabilizes the four-helical oligomeric structure by enhancing electrostatic interaction within the ionic layer. Interruption of the 0 ionic layers disrupts SNARE complex formation. This is a stable interaction, and the zippering and SNARE interaction is thought to provide sufficient force to overcome the energy barrier necessary to fuse membranes (Liu et al., 2006; Grafmüller et al., 2009; Gao et al., 2012; Min et al., 2013).

The characterization of the SNARE motif permitted the discovery of homologous SNARE proteins belonging to the VAMP, syntaxin, or SNAP-25 families and their subsequent localization and expression (Weimbs et al., 1997; Steegmaier et al., 1998; Holt et al., 2006; Arora et al., 2017). Over 40 mammalian SNARE proteins have been annotated to date, and the search for additional SNARE proteins is ongoing (Kloepper et al., 2007; Le and Huynh, 2019; Le and Nguyen, 2019). All SNARE complexes contain the single SNARE motif that contributes an R and three SNARE motifs that each contribute a Q to the ionic 0 layers. SNARE proteins were thus categorized as R-SNAREs and Q-SNAREs (Fasshauer et al., 1998).

**Figure 2:**
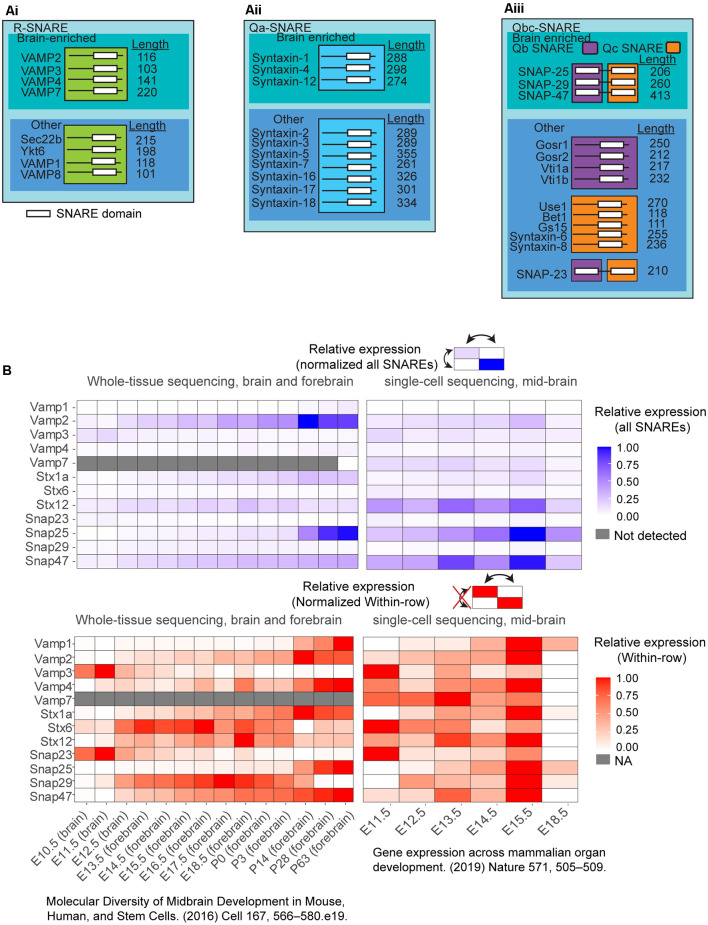
**(A)** Selected list of the classified as Qabc-SNAREs and R-SNAREs and neuronally enriched SNARE expression *in vivo* in the murine brain. SNAREs are classified into: **(Ai)** R-SNAREs; **(Aii)** Qa-SNAREs; and** (Aiii)** Qbc-SNAREs. These SNAREs were further subset into brain-enriched or not brain-enriched. White bars in the illustration represent SNARE domains. **(B)** Expression from two different databases of select SNARE mRNA expression, one from whole-brain and forebrain sequencing, and another database from single-cell sequencing of the mid-brain over multiple developmental time points. SNARE expression was normalized to all SNAREs (blue) or normalized within-row (red) for identification of temporal peak expression for each individual. To compare across all SNAREs (blue), datasets of reads per kilobase of exon model per million mapped reads (RPKM) were filtered for SNARE expression. The expression data were normalized to the highest RPKM among all SNAREs was set to 1, and the lowest RPKM among all SNARES set to 0, and all SNAREs RPKMs were normalized between these two values. To compare SNARE expression within-row (red), the highest RPKM for each SNARE was set to 1, and the lowest RPKM was set to 0 separately for each SNARE.

The R-SNARE family consists of several VAMP-2 family members, which mediate the fusion of different compartments (Figure 2Ai). Exocytic R-SNAREs expressed in the brain include VAMP-1, VAMP-2, VAMP-3, VAMP-4, and VAMP-7. Whereas VAMP-2 is brain-enriched, VAMP-4 and VAMP-7 are broadly expressed and found in multiple tissues (Advani et al., 1998; Wong et al., 1998; Steegmaier et al., 1999; Adolfsen et al., 2004). The Q-SNARE family is divided into three subfamilies, Qa, Qb, and Qc SNAREs, based on the amino acid sequence homology of the SNARE domain (Bock et al., 2001; Kloepper et al., 2007). The syntaxins primarily belong to the Qa subfamily (Figure 2Aii). Syntaxin-1, syntaxin-2, syntaxin-3, and syntaxin-4 are known to mediate exocytosis. Syntaxin-1, syntaxin-4, and syntaxin-12 are brain-enriched, whereas syntaxin-6 and syntaxin-7 are found broadly in most mammalian tissues (Bock et al., 1997; Advani et al., 1998; Hirling et al., 2000; Mullock et al., 2000; Jung et al., 2012). Finally, the SNAP-25 family members are classified as Qb and Qc SNAREs (Figure 2Aiii). The N-terminal SNARE motif (Qb-SNARE) of the SNAP-25 family members are more homologous to each other than the C-terminal SNARE motif (Qc SNARE) of the same protein. The same is also true for C-terminal Qc SNARE motifs. SNAP-23, SNAP-25, and SNAP-47 mediate exocytosis. SNAP-25, SNAP-29, and SNAP-47 are brain-enriched, whereas SNAP-23 is ubiquitously expressed throughout tissue types, including the brain (Arora et al., 2017). Both SNAP-29 and SNAP-47 are widely distributed in the cell, including on synaptic vesicles as well as other intracellular membranes (Holt et al., 2006).

The distinct tissue expression and cellular localization of a large number of SNARE proteins suggested SNARE interactions may impart selectivity to fusion between distinct cellular compartments. Richard Scheller and collaborators however discovered that recombinant or reconstituted R-SNAREs and Q-SNAREs were surprisingly promiscuous, and any combination of Qa, Qb, Qc, and R SNARE formed SNARE complexes *in vitro* (Prekeris et al., 1999). R-SNARE and Q-SNARE combination experiments with VAMP-2, VAMP-4, VAMP-7, VAMP-8, rSec22b; syntaxin-1A, syntaxin-4, syntaxin-12; and SNAP-23, SNAP-25, or SNAP-29 revealed that all 21 combinations of an R-SNAREs and either of the syntaxins and SNAP-25 family members led to thermally-stable complexes. Despite the promiscuity of SNAREs in complex formation *in vitro*, not all SNARE complexes are fusogenic (Parlati et al., 2000, 2002), suggesting additional mechanisms can regulate fusion after SNARE complex formation. If SNAREs provided specificity to which compartments fuse, yet were not selective *in vitro*, this suggests that additional mechanisms must regulate their interactions, such as regulatory proteins or even subcellular localization, and thus likelihood to interact. Although the majority of SNAREs interact *in vitro*, the total number of relevant combinations of SNAREs formed *in vivo* has not been studied in-depth, and whether these combinations of SNAREs change over time is unknown.

### SNARE Roles in Different Stages of Development

#### SNARE Expression Changes Throughout Development

The stereotypical progression of neuronal morphogenesis involves dramatic plasma membrane expansion. Experiments exploiting pharmacological disruption of vesicle trafficking from the Golgi halted the growth of neurons (Craig et al., 1995). Electron microscopy-based studies demonstrated that developing axons and growth cones contained membrane-bound vesicles in the growth cone and cell soma (Igarashi et al., 1997). Subcellular fractionation and subsequent immunoblot analyses demonstrated that these vesicles contained SNAREs (Igarashi et al., 1997). Together, these classic findings suggested that these vesicles undergo SNARE-mediated fusion with the plasma membrane throughout the developing neuron, and this was required for neuronal growth. These and other studies suggested that SNARE-mediated exocytosis delivered the bulk of membrane material to increase the surface area of a growing neuron (Craig et al., 1995; Pfenninger and Friedman, 1993; Horton et al., 2005). Years of research revealed this exocytosis-driven expansion is concomitant with temporal coordination and switches in SNARE protein expression, localization, and function. Interruption of exocytosis or SNARE expression leads to incomplete development or non-viability (Schoch et al., 2001; Washbourne et al., 2002; Winkle et al., 2014; Arora et al., 2017).

To characterize the developmental expression and relative enrichment of different SNARE proteins, we explored two RNA sequencing databases covering time points throughout murine brain development (Cardoso-Moreira et al., 2019; La Manno et al., 2016; Figure 2B). VAMP-2 was the highest expressed R-SNARE, increasing in expression late into development, whereas SNAP-25 and SNAP-47 were the most enriched Qbc-SNAREs, and syntaxin-1 and syntaxin-12 the highest expressed Qa-SNAREs. After birth, SNAP-25 and VAMP-2 expression increased two-fold over other SNAREs, suggesting these SNAREs had a significant role in mature neuronal function (Figure 2B, P0-P63). At E18.5 in the single-cell sequencing database, the majority of genes in the midbrain have lower expression compared to E15.5, differing from the trend in whole-brain sequencing. This observation remains to be explored. The heterogeneity in developmental time points in which different SNARE expression peaked suggests that each SNARE may play a distinct role in neuronal development.

Several SNARE proteins are implicated in developmental plasma membrane expansion, with different SNARE complexes involved depending on the extracellular environment, morphogens, and state of morphogenesis. For example, neuritogenesis *in vitro* is context-specific. Poly-D-Lysine (PDL) is an inert, synthetic adhesive polymer that promotes cell adhesion to tissue culture-treated plastic and glass surfaces without activating known adhesion receptors. In cortical neurons grown on PDL-coated surfaces, the outgrowth of neurites was tetanus neurotoxin (TeNT) sensitive at early stages. TeNT cleaves VAMP-1 and VAMP-2 and prevents their assembly into SNARE complexes. The TeNT sensitivity of neuritogenesis, therefore, suggested neurite outgrowth required VAMP-2 (and possibly VAMP-1)-mediated exocytosis. This is consistent with the high expression of VAMP-2 relative to other R-SNAREs at this developmental time point (Figure 2B). Laminins are extracellular matrix ligands that bind specific integrin heterodimers, syndecan, and α-dystroglycan, and are the major component of the basal lamina and are found at specific areas in the brain. When neurons were grown on laminin, neurite outgrowth was no longer TeNT-sensitive and was instead mediated by TeNT-insensitive VAMP(TI-VAMP), also known as VAMP-7 (Osen-Sand et al., 1996; Sosa et al., 2006; Gupton and Gertler, 2010). Laminin engagement of integrin receptors was suggested to activate distinct signaling pathways and SNARE proteins, such as VAMP7(Gupton and Gertler, 2010). During neuritogenesis of cortical neurons on PDL, we recently found that VAMP-2-mediated exocytic events localized in a spatially random fashion and occurred at a higher frequency than at later points of neuronal morphogenesis, after a presumed axon had formed (Urbina et al., 2018). The rapid and random occurrence presumably inserted maximal membrane material before the axon was specified. The context-dependent usage of VAMP-2 or VAMP-7 suggested that neurons utilized specific VAMPs based on the extracellular environment. Whether other SNARE proteins function in a context-dependent fashion has not been explored. For example, if a neuron encounters a gradient of laminin or other morphogens, does R or Q-SNARE utilization change based on a threshold?

After neuritogenesis, a subsequent outgrowth of neurites from the polarized hippocampal neuron is mediated by VAMP-7, but not VAMP-2 (Martinez-Arca et al., 2001; Osen-Sand et al., 1996). SNAP-25, syntaxin-1, and syntaxin-12 were also reported necessary for axon outgrowth (Igarashi et al., 1996; Hirling et al., 2000; Zhou et al., 2000; Kabayama et al., 2008). *In vivo*, VAMP-7 and syntaxin-12 expression peaked early-to-mid brain development (~E13.5-E18.5), whereas syntaxin-1 and SNAP-25 expression increased substantially during these time points (Figure 2B). Using acute treatment of botulinum neurotoxin A(BoNT/A) to cleave SNAP-25 resulted in the loss of neurite outgrowth, whether neurons were grown on laminin or PDL(Osen-Sand et al., 1996). Curiously, in contrast to cleavage with TeNT or BoNT, genetic deletion of either murine *Vamp*2 or *Snap25* did not inhibit neuritogenesis *in vitro*, and neurite outgrowth proceeded (Schoch et al., 2001; Washbourne et al., 2002). *Snap25*^−/−^ cultured cortical neurons have reduced dendritic length, and soma area, but similar axonal lengths compared to wildtype neurons at day 4 *in vitro* (Arora et al., 2017). This apparent disagreement suggested that the SNAREs compensated following the genetic deletion of either *Vamp2* or *Snap25*, whereas this did not occur sufficiently following acute cleavage and disruption of SNARE protein function. For example, experimental evidence suggested VAMP-7 can substitute for VAMP-2, and overexpression of SNAP-23 or SNAP-29 rescued SNAP-25 deletion phenotypes *in vitro* (Winkle et al., 2014; Arora et al., 2017).

The signals that initiate axon specification and outgrowth are not fully understood; however, the localization of insulin-like growth factor 1 (IGF-1) receptor in the plasma membrane of hippocampal neurons to a single neurite, followed by phosphatidylinositol (3,4,5)-triphosphate (PIP3) accumulation at the growth cone of that neurite were found to be critical events (Sosa et al., 2006). The localization and insertion of the IGF-1 receptor were mediated by another set of SNAREs: VAMP-4, syntaxin-6, and SNAP-23 (Grassi et al., 2015). IGF-1 receptor insertion was suggested to promote a positive feedback loop, eventually specifying the single neurite as the axon. Following axon specification, the growth cone navigates toward targets of innervation by sensing extracellular cues. Several studies have indicated SNARE-mediated fusion was critical for axon guidance (Tojima et al., 2007; Zylbersztejn et al., 2012; Tojima and Kamiguchi, 2015). For example, VAMP-2 was required for attractive responses to nerve growth factor (Tojima et al., 2007; Akiyama and Kamiguchi, 2010). TeNT treatment or genetic deletion of VAMP-2 nullified the turning response of a growth cone in a repulsive dose of sema3A. Similarly, SNAP-25 and syntaxin-1 were involved in the axon turning response to the guidance cue netrin-1. Netrin-1 induced coupling of its receptor, Deleted in Colorectal Cancer (DCC) to syntaxin-1 (Cotrufo et al., 2012). Abolishing syntaxin-1 function through syntaxin-1-shRNA knockdown or BoNT/C1 treatment (which cleaves both syntaxin-1 and SNAP-25), disrupted axon turning, whereas BoNT/A cleavage of SNAP-25, or TeNT cleavage of VAMP-2, did not disrupt turning. The lack of phenotype with acute SNAP-25 or VAMP-2 disruption suggests that syntaxin-1 may form SNARE complexes with additional SNAREs. Indeed, netrin-1 treatment of murine cortical neurons increased the frequency of both VAMP2 and VAMP7-mediated exocytosis (Winkle et al., 2014). Further experiments determined the spatial distribution of exocytosis on the growth cone was important in the turning response. Most guidance cues instruct growth cone turning *via* asymmetric Ca^2+^ signals, with a higher Ca^2+^ concentration on the side of the growth cone facing the source of the cues, regardless of whether the cues are attractive or repulsive (Gomez and Zheng, 2006). The repulsive cue sema3A suppressed VAMP-2 mediated exocytosis. Tojima et al. (2011) discovered that using local photo-uncaging of Ca^2+^ on one side of a growth cone increased local VAMP-2-mediated exocytosis and subsequent growth cone turning. Asymmetrical VAMP-2-mediated exocytosis was suggested to turn the growth cone toward the side of the highest exocytic release.

#### The Mature Neuron

After the growth cone reaches its target destination, synaptogenesis begins. SNAP-25 is recruited to presynaptic sites, where it participates with VAMP-2 and syntaxin-1 in synaptic vesicle release (Südhof, 2004). SNARE proteins are best characterized for their role in synaptic transmission. Evoked synaptic response is non-existent in neurons cultured from mice lacking VAMP-2 or SNAP-25 (Sørensen et al., 2003). *Snap-25*^−/−^ cultured neurons eventually die before synaptogenesis (Arora et al., 2017). This may depend on survival factors, as lower densities of cultured *Snap-25*^−/−^ hippocampal neurons die within 2–3 days *in vitro*, whereas higher densities died only after 7 days *in vitro*, after forming immature synapses (Washbourne et al., 2002; Arora et al., 2017). Of the neurons that survived to day 8 *in vitro* (~2%), they have fewer synapses and dendrites compared to *Snap-25^+/+^* neurons. Expression of SNAP-23 or SNAP-29 in SNAP-25 deficient neurons rescued dense-core vesicle release; however, expression of SNAP-47 did not rescue this defect, suggesting that although SNAREs can substitute for each other in SNARE complexes, they were not always capable of restoring the functional role of another SNARE family member (Washbourne et al., 2002; Arora et al., 2017). These results and their contrast to acute inhibition of SNAREs with BoNT or TeNT suggest that compensatory actions of other SNARE proteins occurred following genetic deletion, or that acute inhibition vs. genetic deletion triggered a difference in growth and survival cues. At the post synapse, SNAP-25, and likely VAMP-2 are involved in the exocytosis-dependent insertion of NMDA receptors (Lau et al., 2010). In addition to the well-studied SNAP-25, other Qbc-SNAREs are also important in synaptic function. SNAP-23 mediates NMDA receptor insertion in postsynaptic dendritic spines, a marker of synaptic plasticity, whereas overexpression of SNAP-29 inhibited synaptic vesicle fusion (Hunt and Castillo, 2012; Wesolowski et al., 2012; Cheng et al., 2013). SNAP-47 regulated the release of BDNF, which modulated long-term potentiation and spine regulation in hippocampal neurons (Shimojo et al., 2015).

Although SNAREs are necessary for evoked synaptic release and proper neuronal development *in vitro*, one striking result was the lack of discernible morphological phenotypes when critical SNARE genes were deleted globally during development *in vivo*. In both VAMP-2 and SNAP-25 deficient mice, fetuses were born with a rounded body shape distinct from wildtype, but no gross brain malformations. The mice lacked synaptic transmission, however, and died immediately following birth, presumably due to paralysis of the lungs (Washbourne et al., 2002; Arora et al., 2017). Histological examination of stained paraffin brain sections from E17.5 and E18.5 *Snap-25*^−/−^ mice showed no evidence of cellular defects (Washbourne et al., 2002). Although there has not been a careful examination of neuronal morphology in these knockout mice, axon and dendrites were present. Based on the phenotype associated with acute inhibition of SNAP-25 with BoNT-A *in vitro*, this suggested compensation for fundamental SNARE exocytosis occurs in this developmental time frame, whereas specialized roles for synaptic transmission cannot be similarly compensated for. Immunocytochemical stain for tyrosine hydroxylase, a marker for mature catecholaminergic neurons, demonstrated a normal pattern of immunoreactivity in the *Snap-25*^−/−^ brainstem. Thalamocortical axon projections, traced using carbocyanine dye from E17.5-E19, were unaffected by the deletion of *Snap-25* (Molnár et al., 2002). In *Vamp-2* null mice, analysis of brain sections revealed no neurodegeneration or other gross changes to brain structure (Schoch et al., 2001). Although a careful examination was not performed, neurons lacking VAMP-2 had normal-appearing neurons with dendrites and axons, again suggesting that compensation *via* other SNARE proteins occurred during the developmental time window (Schoch et al., 2001). The context-dependence of SNAREs and their varying expression levels during development suggest that SNAREs may perform multiple roles throughout development. One question in the field is whether SNARE-composition changes during different stages of development.

### Sites of Membrane Growth

Where the material is added to the plasma membrane during neurite outgrowth continues to be an outstanding question. Early attempts to address this conundrum ended with mixed results; both the cell body and distal growth sites, such as the growth cone, were proposed as sites of membrane addition, and both locations have garnered support and contradictory evidence. Despite this ambiguity, membrane addition at the sites of growth has become the favored hypothesis for where surface area expansion is occurring (Pfenninger, 2009; Tojima and Kamiguchi, 2015).

#### Evidence for the Growth Cone

The debate surrounding where membrane insertion occurs in growing neurons was introduced by *Dennis Bray*. In classic experiments, Bray observed that glass particles resting on rat sympathetic neurons remained stationary relative to the soma as the neuron elongated (Bray, 1970). He proposed that membrane insertion occurred primarily at the distal ends of growth. Following the fate of a radio-labeled phospholipid precursor, [^3^H]glycerol, in pulse-chase experiments in conjunction with electron microscopy-based visualization of silver grains marking incorporation sites of radioactive material demonstrated that pulsed lipids initially accumulated in ER and vesicles, and subsequently at distal ends of growing axons (Pfenninger and Friedman, 1993). An alternative approach used to suggest sites of membrane insertion involved following the fate of transmembrane proteins, which were presumably trafficked to and inserted at sites of membrane growth. The expression of an exogenously tagged transmembrane protein CD8a, followed by fixation and immunolabeling of CD8a, showed that CD8a localized in the growth cone 8-h post-transfection, and a smaller amount localized in the cell body (Craig et al., 1995). If protein transport was blocked using Brefeldin A treatment, CD8a was not detected in the growth cone and rather accumulated in the soma. Whereas authors interpreted these results to suggest the addition of new membrane occurs at sites of growth, each of these early experiments suffers from being indirect methods of determining where a new surface area is added.

#### Evidence for the Soma

Another line of experiments, however, suggested that the soma was the primary site of plasma membrane expansion. Popov et al. (1993) directly labeled the lipid bilayer of *Xenopus* spinal neurons by application of fluorescent lipids. After local infusion of fluorescent lipids near the soma, the authors detected anterograde transport of the exogenous fluorescent lipids using time-lapse microscopy. This anterograde transport persisted regardless of where along the axon the lipid dyes were infused, suggesting that membrane inserted at the soma and flowed toward the growth cone. Although Pfenninger and Friedman’s conclusions were at odds with Popov et al, without the capability of following where vesicle fusion with plasma membrane inserts membrane material, all these methods only provided snapshots that could lead to different interpretations depending on the context. SNARE localization and early tracking of fusion events added support for SNARE-mediated fusion as a driver of membrane insertion. VAMP7-containing vesicles translocated from the soma to become enriched in the growth cone, where they then fused (Burgo et al., 2012). Evidence indicated that VAMP2 was found throughout the cell and VAMP-2-mediated exocytosis also occurred at the growth cone periphery, including at filopodia (Sabo and McAllister, 2003; Tojima et al., 2007; Ros et al., 2015). Recent improvements in imaging, such as the use of pH-sensitive probes, provided new ways to visualize where membrane insertion occurs more directly. Using vSNAREs lumenally labeled with pHluorin, a pH-sensitive GFP allowed direct visualization of exocytic events. The fluorescence of pHluorin is quenched in the acidic environment of the vesicle lumen. However, upon opening a fusion pore between the vesicle and plasma membrane, protons rapidly escape the vesicle lumen, and the neutralized pH promotes rapid unquenching and fluorescence of pHluorin, as a readout of fusion pore opening (Miesenböck et al., 1998). In conjunction with Total Internal Reflection Fluorescence (TIRF) microscopy, which specifically illuminates the basal plasma membrane of the neuron, and an unbiased, automated image analysis method, we recently reported that the majority of constitutive VAMP2-mediated exocytosis occurred in the soma in stage one and stage two neurons, with a much smaller proportion occurring in distal neurites and growth cones (Urbina et al., 2018). By the time neurons reached stage three, VAMP-2- mediated exocytic events were evenly distributed between soma and neurites but were still rarely observed in growth cones. In contrast to VAMP-2, VAMP-7-pHluorin mediated exocytic events were less frequent and sometimes undetected, suggesting that VAMP-2-mediated exocytosis primarily accounted for exocytic membrane addition during these early developmental stages. This result was consistent with cortical neuritogenesis being VAMP-2 but not VAMP-7 dependent (Gupton and Gertler, 2010). During this developmental stage, VAMP-2 mRNA expression increased (Figure 2B), also consistent with an important role for VAMP-2 during neuritogenesis. We detected an average of two VAMP2-pHluorin events per growth cone per minute, in agreement with other reports (Ros et al., 2015; Urbina et al., 2018). In contrast, we detected 8–10× the number of exocytic events in the soma in a similar period. In addition to quantifying rates and spatial location of individual exocytic events, we measured the size of non-coated vesicles (presumably exocytic) and clathrin-coated vesicles by EM, the size of neurons at multiple developmental time points, as well as the rate of endocytosis, to establish a model of cell growth. From these data and modeling, we concluded that the rate of exocytosis in growth cones was orders of magnitude less than was required for the growth of the neuron. In contrast, exocytosis in the soma, not the growth cone, provided more than sufficient membrane to account for membrane expansion concomitant with the growth of developing neurons. Although this system used overexpression of VAMP-2, VAMP-2-pHluorin marked the vast majority of VAMP2 containing vesicles, suggesting it accurately represented the population of fusing events (Urbina et al., 2020). Our results called into question the traditional view that membrane was preferentially inserted at sites of neurite extension. Prior results demonstrating the accumulation of transmembrane proteins at the growth cone, which appeared to contradict our findings, may suggest that specialized vesicles are primarily trafficked to the growth cone for signaling and chemotrophic-induced asymmetric exocytosis required for turning, whereas the bulk of membrane material was inserted at the soma. Alternatively, the soma and growth cone may both be sites of growth and insertion, with membrane preferentially removed from the soma, but not the growth cone.

#### A Point of Tension

Membrane tension, defined as the force per unit length acting on a cross-section of the plasma membrane, regulates the frequency and the localization of exocytic events (Gauthier et al., 2011; Diz-Muñoz et al., 2013; Bretou et al., 2014; Wen et al., 2016). For example, mechanical tension can alter the organization of the submembranous actin network, which may regulate vesicle access and fusion (Wen et al., 2016). Mechanical forces on the growth cone, originating from adhesions to the extracellular environment, as well as cytoskeleton dynamics, induce changes in membrane tension, and influence growth rates. For example, the location of sites of growth was altered by mechanical forces encountered by the neuron (Zheng et al., 1991). An experimentally-induced towing force transduced by pulling a glass needle attached to the growth cone increased axon elongation rate by 1.5 μm/h/μdyne, with some neurons exhibiting a 350 μm/h increase in growth rate (Zheng et al., 1991). Neurons were labeled with polyethyleneimine-treated microspheres, and the position of microspheres along the axon was recorded. Without towing, the microspheres remained stationary relative to the soma during neurite outgrowth. However, in pulled neurites, all the microspheres along the axon increased in distance from the soma as well as from each other, suggesting that membrane insertion occurred along the entire length of the neurite. This suggested that the spatial location of exocytic growth was context-dependent, and regulated by membrane tension.

The ability of local changes in membrane tension to regulate membrane insertion distally remains unclear, as the capability for the plasma membrane to propagate tension is not fully understood. The fluid-mosaic model, which posits the plasma membrane as liquid-like, suggests that membrane flow transmits local changes in membrane tension across the cell in milliseconds, providing a long-range signaling pathway. The fluid-mosaic model suggests tension propagates across the entire surface of artificial membranes, based on the capacity for fluorescently-tagged proteins to freely diffuse in both artificial bilayers and intact cells (Mouritsen and Bloom, 1984; Sperotto et al., 1989; Kusumi et al., 2005). In contrast, the Cohen lab proposed that the density of transmembrane proteins influences gel-like properties of the plasma membrane and that tension does not propagate across the plasma membrane, but only locally activates tension-related signaling (Shi et al., 2018). The gel-model of the plasma membrane has wide implications for neurons. If membrane addition is primarily added to the soma, the gel-model of the plasma membrane suggests the added material generates a pushing force to extend axons during growth. The potential pulling force of the growth cone or pushing force of the membrane at the soma could, in effect, change exocytic fusion dynamics. The potential influence of membrane tension or fluidity on the spatial location and rates of exocytosis suggest that a different cell type, a different substrate, or indeed the variety of stiffnesses of substrates found in the brain may orchestrate entirely different spatial organization to exocytosis. An outstanding question in the field is, then, whether *in vitro* studies accurately reproduce conditions to define where membrane addition is occurring *in vivo*, and whether different neuronal types add membrane at different spatial locations based on differences in tension in the extracellular environment.

### Modes of Fusion

**Figure 3:**
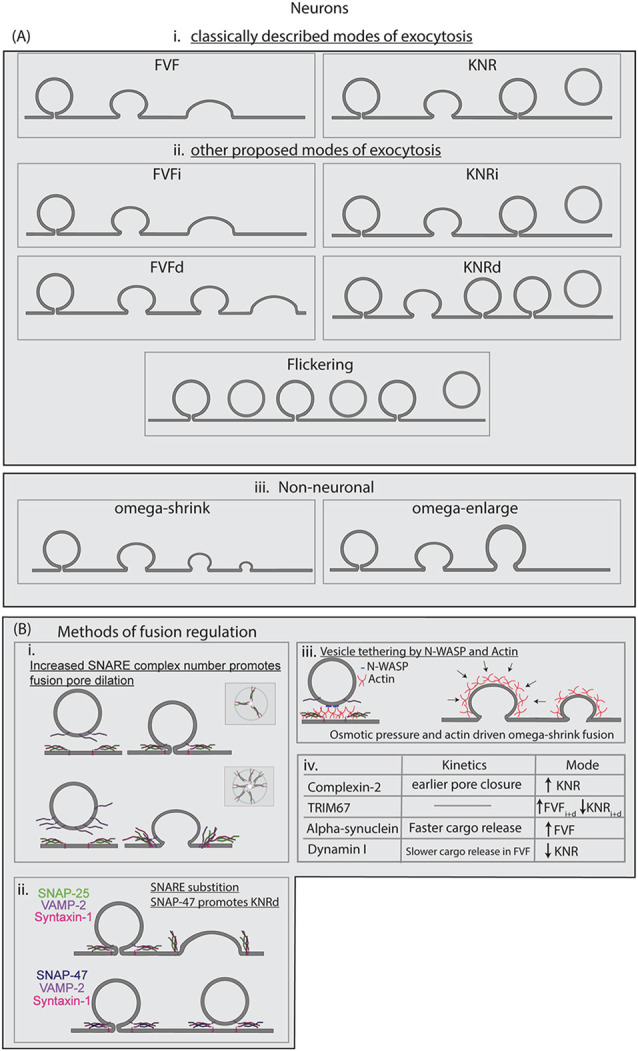
**(A)** Modes of exocytosis described in the literature for neuronal and non-neuronal cells. Full-vesicle fusion (FVF) and KNR were the first proposed modes of exocytosis. Recently we proposed the subclasses of FVFi, FVFd, KNRi, and KNRd to best describe the heterogeneity observed in developing cortical neurons. Flicker fusion has been observed at hippocampal synapses, resulting in the vesicle retreating from the membrane. In neuroendocrine cells, omega-shrink and omega-enlarge have been described as the primary modes of exocytosis observed. **(B)** Known regulations of SNARE-mediated fusion are described, including regulation of the modes of exocytosis. **(i)** The number of SNARE complexes at the fusion pore increases the probability of fusion pore opening and accelerates fusion pore expansion. **(ii)** SNARE-substitution can alter fusion dynamics, such as SNAP-47 substituting for SNAP-25. SNARE complexes made up of different SNAREs have distinct kinetic profiles. **(iii)** Spatial segregation or aggregation, such as linking of vesicles to the plasma membrane by N-WASP, can regulate fusion. Actin dynamics also regulate fusion dynamics, and actin as well osmotic pressure is suggested to drive FVF as well as omega-shrink fusion in neuroendocrine cells. **(iv)** A limited list of proteins suggested altering modes of fusion and the kinetics of SNARE-mediated fusion in neurons.

Our model of membrane growth concerning exocytosis and endocytosis in developing neurons suggested exocytosis added superfluous membrane material for neuronal morphogenesis. One caveat of the model however was that it assumed that all exocytic events inserted the entirety of the vesicular membrane into the plasma membrane (Urbina et al., 2018). However, two modes of exocytosis are described prominently in literature, each of which has different consequences for plasma membrane expansion and cargo secretion. In this section, we review the history of findings regarding modes of fusion and their repercussions on neuronal growth. Visualization of the fusion pore by electron microscopy initiated a controversy regarding modes of fusion, as the initial results were interpreted in different ways. In one camp, Ceccarelli et al. (1972) proposed that vesicle fusion involved the opening of a small fusion pore between the vesicle and plasma membrane. This was followed by rapid pore closure at the site of fusion, without full dilation and collapse. This model referred to primarily as kiss-and-run (KNR), originated from the electron microscopic observation of the uncoated omega-shaped membrane profile with a narrow neck connected to the plasma membrane at the active zone of the frog neuromuscular junction (Figure 3Ai). Meanwhile, other electron microscopy-based studies of the frog neuromuscular junction by Heuser and Reese (1973) demonstrated omega-shaped profiles with narrow or wide necks, as well as areas interpreted as a fully-dilated fusion pore (Heuser and Reese, 1973; Heuser, 1989). They suggested a model in which fusion pore opening was followed by fusion pore dilation and the full collapse of the vesicle membrane into the plasma membrane. This was followed by membrane retrieval at sites distal to fusion (Figure 3Ai). This mode of fusion has different names throughout literature, including full-collapse fusion and full fusion, but we shall refer to it as full-vesicle fusion (FVF). FFV events add material to the plasma membrane, expanding the neuronal surface area, whereas KNR events do not. FVF events allow the quantal release of all cargo, whereas KNR events allow partial cargo release. These fundamental distinctions suggest that KNR and FVF are poised to play diverse roles in development and at the synapse. During neuronal development, FVF can add plasma membrane material and insert transmembrane receptors. FVF can also add membrane to the synapse, expanding surface area. Therefore, to maintain a consistent synapse size, compensatory endocytosis must retrieve excess plasma membrane (Heuser and Reese, 1973). KNR does not provide for developmental plasma membrane expansion but may enable more rapid and economical vesicle recycling at the synapse. Second, the narrow fusion pore during KNR may limit the rate of transmitter discharge, resulting in a more tuneable synaptic response (Stevens and Williams, 2000). Switching between KNR and FVF provides a mechanism to regulate membrane addition and cargo release, as well as regulate synaptic transmission.

The controversy of modes of fusion both at synapses and in non-neuronal cell types continued for decades, in which evidence accumulated to support multiple fusion modalities (Stevens and Williams, 2000; Rizzoli and Jahn, 2007). The existence of multiple exocytic modes at the synapse remained controversial as multiple vesicles fuse near-simultaneously in a diffraction-limited area, and thus single-fusion event conclusions were often extrapolated from the aggregate of a population of fusing vesicles. Evidence using imaging and electrophysiological approaches to track exocytosis at high spatial and temporal resolution suggested that low frequency or low-release probability fusion events were primarily KNR (Gandhi and Stevens, 2003; Harata et al., 2006). Using FM dyes, Aravanis et al. (2003) demonstrated that vesicles loaded with FM1–43 dye exhibited partial or complete dye loss during stimulation. Using the hydrophilic FM1–43 quencher bromophenol blue revealed vesicles retained part of the dye after fusion, consistent with KNR. Using VAMP2-pHluorin, Harata et al. (2006) investigated the time course of exocytosis fusion and fluorescence decay. They found that there was a population of fast-fluorescing events that exhibited a rapid decrease in fluorescence after fusion pore opening distinct from that of average exocytic fusion event decay. This rapid decrease in fluorescence was slowed through the addition of the pH buffer TRIS, which slowed vesicle re-acidification, suggesting these events were KNR (Harata et al., 2006). Although the challenges of imaging individual fusion events at the synapse remain a hurdle, the evidence for KNR has continued to grow.

Electrophysiological evidence supporting distinct FVF and KNR fusion have also been obtained in neuroendocrine cells. For example, membrane capacitance, measured by electrophysiology techniques, is proportional to membrane area, and thus offers a readout of membrane expansion. As such, membrane capacitance increases when a vesicle fuses with the plasma membrane and increases the plasma membrane surface area. A sustained increase in capacitance following fusion indicates FVF; capacitance “flickers,” in which transient capacitance increases occurred, suggests KNR. Both types of events were detected in neuroendocrine cells (Fernandez et al., 1984). Subsequent capacitance measurements suggested the fusion pore diameter remained under 3 nm for a variable period before expanding in the majority of events, presumably FVF, whereas a smaller proportion of events never fully dilated but instead closed, presumably performing KNR (Albillos et al., 1997).

Imaging data of single vesicle fusion events supported the existence of KNR and FVF in astrocytes and in neurons outside of synapses. Using VAMP2-pHluorin and TIRF microscopy, Bowser and Khakh discerned the kinetic profiles of individual fusion events in astrocytes. They differentiated FVF events by the diffusion of VAMP2-pHluorin away from the site of fusion, whereas KNR events were differentiated by the disappearance of fluorescence without diffusion (Bowser and Khakh, 2007). Exocytic events that did not exhibit VAMP2-pHluorin diffusion had half-lives that were sensitive to changes in extracellular pH and buffering capacity, suggesting that the decay of fluorescence was attributable to vesicle re-acidification. Our results corroborated VAMP2-pHluorin diffusion in FVF and reacidification in KNR in developing neurons before synaptogenesis (Urbina et al., 2020). By simultaneously using VAMP2-pHluorin and VAMP2-tagRFP, we followed the fate of VAMP2-containing vesicles before, during, and after fusion. This revealed distinct modes of fusion exhibiting diffusion of both fluorophores (FVF) or retaining a visible VAMP2-tagRFP containing vesicle after the loss of fluorescence of VAMP2-pHluorin fluorescence (KNR). With knowledge of these distinct fusion modes, we updated our model of plasma membrane expansion to reflect the proportion of exocytic events that would not add membrane (Urbina et al., 2020). Although our original model overestimated the amount of surface area added by exocytosis, our new model, which incorporated the mode of fusion, reflected measured surface area increases of neurons *in vitro* more accurately. Single vesicle fusion events also occur in mature neurons outside the synaptic transmission. Richards combined imaging and electrophysiology to reveal that vesicles containing AMPA receptors at the postsynapse fused primarily through FVF, whereas vesicles containing NMDA receptors fused primarily using KNR (Richards, 2009). Therefore, fusion mode can be specific to distinct vesicle populations with different functional roles and cargo. With sufficient evidence that KNR and FVF are major modes of fusion, the relative contribution of each mode to neuronal development and synapse function remains an important question in cell biology.

#### Beyond Two Modes of Fusion

The heterogeneity of fusion pore behavior and the myriad regulatory proteins that govern fusion pore kinetics raised the question of whether exocytic events can be described by only two fusion modes. For example, additional modes of fusion have been argued to exist in developing neurons, neuroendocrine cells, and at dopaminergic synapses (Figures 3Aii,iii; Stevens and Williams, 2000; Staal et al., 2004; Shin et al., 2018; Urbina et al., 2020). These modes include flickering fusion, shrink-fusion, and subclasses of FVF and KRN. In flickering fusion, the fusion pore is suggested to open and close transiently before the vesicle eventually retreats from the plasma membrane. This mode has been observed primarily in neuroendocrine cells, although evidence suggests it may also occur at synapses (Staal et al., 2004; Wightman and Haynes, 2004; Stratton et al., 2016; Bao et al., 2018). Utilizing pH-sensitive fluorescent proteins within the vesicle lumen, coupled with rapid switching of the extracellular pH *via* microfluidics allowed the detection of opening and closing of fusion pores. In this experiment, fluorescence coinciding with a neutral extracellular pH indicated the opening of a fusion pore. A vesicle with an open fusion pore flickered with the pH change, whereas a sealed vesicle did not change fluorescence. Using this system, a third fluorescence behavior was observed: irregular switching between flickering and stable fluorescence. This observation suggested a population of vesicles with fusion pores that repeatedly opened and closed, hence the name flickering fusion. In shrink-fusion, the omega-shape of vesicles shrinks due to the osmotic pressure of the cell without fusion pore dilation, and enlarge-fusion, in which the omega-profile of the fused vesicle increased in size after the opening of the fusion pore (Shin et al., 2018, 2020). More recently, Wu et al imaged over 300 fusion events in neuroendocrine cells using stimulated emission depleted (STED) microscopy and remarkably found no events that fully dilated until vesicle collapse (Shin et al., 2018). The authors found instead that fusion pore size positively correlated with vesicle size, which ranged from 180 to 720 nm in diameter. Although fusion pore size was variable, full dilation was not observed, and rather fusion pore opening was followed by a shrinking omega-shaped profile. Of note, this variation on fusion mode adds material to the plasma membrane. Whether this omega shrink fusion happens in other cell types or at the synapse is an open question.

Diverse fusion behavior was also observed in developing neurons before synaptogenesis. As described above, FVF and KNR can be differentiated based on VAMP2-pHluorin diffusion, pH sensitivity, or the behavior of a pH-sensitive VAMP2-RFP. Recently we identified distinguishable subclasses of FVF and KNR in developing cortical neurons using an unbiased classification of the fluorescence profiles of VAMP2-pHluorin fluorescence after fusion pore opening (Urbina et al., 2020). A subset of exocytic events exhibited an immediate (i) decay of VAMP2-pHluorin fluorescence after fusion pore opening. Both FVF and KNR events were observed with this behavior and subsequently termed FVFi and KNRi. FVFi and KNRi are analogous to “classical” FVF and KNR fusion as described in the literature. A previously unappreciated subset of events exhibited a plateau in VAMP2-pHluorin fluorescence after fusion pore opening, indicating a delay (d) in fluorescence decay after fusion pore opening, and thus a delay in the vesicle fusion process. Some of these delayed events eventually proceeded to FVF (FVFd), and some proceeded to KNR (KNRd). The existence of these delayed events suggested mechanisms were capturing an open fusion pore before the vesicle proceeded to a final vesicle fate of FVF or KNR. The distribution of these four modes of exocytosis between developing neurons was remarkably consistent. The heterogeneity of cell type-specific fusion behaviors may indicate that classic FVF and KNR are the predominant modes of fusion only in a subset of cell types. The number of different modes, how fusion modes differ between cell types, and the mechanisms that govern modes of fusion are open questions in the field.

### The Fusion Pore: Kinetics, Dynamics, and Models of Formation

#### The Fusion Pore: Expansion and Contraction

The mode of fusion is necessarily dictated by the fate of the fusion pore. Analyzing the fate of the fusion pore has remained controversial due to the enigmatic nature of fusion events, which are spatially small and temporally rapid. Reconstituted systems allow examination of how fusion proceeds using the minimal machinery required. in vitro experiments of liposomal fusion with varying densities of reconstituted SNARE proteins suggested a critical number of SNARE complexes were required to open and dilate the fusion pore and accomplish content mixing (Lu et al., 2005). Similarly, cell fusion assays using nanodiscs, synthetic lipoprotein particles that contain a small (~17 nm diameter) circular lipid bilayer with differing numbers of SNAREs, suggested two SNARE complexes were required to open a fusion pore, but seven SNARE complexes were required to fully dilate the pore (Shi et al., 2012). This number of SNARE complexes required for fusion was consistent with predictions from the Karatekin and O’Shaughnessy lab supporting their biophysical model of SNAREpin repulsion as an explanation for fusion pore expansion (McDargh et al., 2018).

With more complex reconstitution assays, the heterogeneity of fusion modes emerged. Using nano-discs and HeLa cells, Wu et al. (2016) found two fates for the fusion pore after opening: fully dilated or closed. In this assay, a pipette was sealed onto a HeLa cell expressing flipped Q-SNAREs, with the active SNARE domains facing the extracellular space. Nanodiscs with R-SNAREs were then flooded into the pipette, and fusion events were probed by direct-voltage electrophysiology. The majority of events measured in this assay were FVF. The prevalence of FVF over KNR is consistent with biophysical models, which suggested FVF be energetically favorable over KNR (Mostafavi et al., 2017; Risselada et al., 2014; Wu et al., 2017). Bao et al. (2018) detected rapid opening and closing of fusion pores in a reconstituted fusion assay through electrophysiological spikes using nanodiscs. As the number of SNAREs per nanodisc increased, the probability of the fusion pore remaining open increased, and fusion pore dilation increased. The recapitulation of KNR-like, FVF-like, and flickering fusion suggested the number of SNAREs available for fusion regulated the mode of fusion in SNARE reconstitution assays.

#### Regulation of the Fusion Pore

Whereas these assays have reconstituted a variety of fusion pore behavior, such as dilation consistent with FVF, closure consistent with KNR, and rapid opening and closing consistent with flickering, other variations of fusion such as omega shrink have yet to be reconstituted. Not only is the outcome of the fusion pore distinct in different systems, the kinetics of fusion pore opening, dilation, and closing between a liposomal fusion assay event and a fusion event in a cell can be orders of magnitude apart. The difference in fusion pore behaviors in an artificial system compared to cells may be explained by the lack of regulatory proteins as well as differences in the cellular and extracellular environments that fusion takes place compared to *in vitro*.

Sufficient numbers of SNAREs, the minimal machinery for fusion in liposomes, promoted full dilation of the fusion pore, suggesting that additional regulatory mechanisms constrain the fusion pore in the cellular context (Wu et al., 2017; Bao et al., 2018). In nano-disc reconstitution assays, the probability of pore dilation increased with the number of SNARE complexes, suggesting that SNARE density and availability for fusion regulated exocytic fusion (Figure 3Bi; Bello et al., 2016; Wu et al., 2017). Using super-resolution microscopy, Yang et al. (2012) showed that SNAP-25 was clustered in microdomains on the plasma membrane of PC-12 cells and that R-SNARE containing vesicles were trafficked to locations with a lower density of SNAP-25 than the surrounding area, suggesting spatial distancing as a method of regulating fusion. Evidence from Bowser and Khakh (2007) and Urbina et al. (2020), however, suggested the concentration of VAMP-2 was not different between vesicles that fuse by FVF or KNR in astrocytes and developing neurons. These assays relied on measuring the fluorescence of VAMP2-pHluorin, however, and may not be a suitable proxy to capture differences in SNARE complex numbers.

In addition to SNARE complex numbers, SNARE complex composition may play an important role in heterogeneity in fusion in cells. SNARE proteins can substitute for each other in complex and influence fusion kinetics. However, the role that SNARE complex composition plays in the regulation of exocytosis has not been explored until recently. Most artificial assays have utilized SNAP-25, VAMP2, and syntaxin-1 to reconstitute fusion and explore the dynamics of the fusion pore. In such assays, SNAP-47 competed with SNAP-25 to form stable SNARE complexes with VAMP2 and syntaxin-1(Holt et al., 2006). Although capable of fusing proteoliposomes, SNAP-47, VAMP2, syntaxin-1 complexes exhibited slower fusion than SNAP-25, VAMP2, syntaxin-1 complexes. Our recent work in developing neurons suggested SNAP-47 was involved in VAMP2-mediated exocytic fusion at the plasma membrane of developing neurons, and that SNAP-47 containing SNARE complexes may regulate fusion pore kinetics and bias the mode of exocytosis toward KNR events (Figure 3Bii; Urbina et al., 2020). If heterogeneous SNARE complexes existed in the same location spatially, then a combinatorial effect on fusion behavior may emerge, where the kinetics and mode of the fusion are influenced not only by the number of SNARE complexes but also the composition of each of those SNARE complexes. This effect is unexplored in current literature, and further studies are needed to determine the composition of the SNARE complex surrounding fusion pores in cells, and whether heterogeneity in SNARE composition may regulate kinetics and mode of fusion.

An important consideration for comparisons between *in vitro* and artificial systems is that the lipid and protein composition of the plasma membrane, as well as tension propagation, is substantially different from simplistic liposomes, and the proteins that may regulate the opening and kinetics of the fusion pore are not included in most *in vitro* fusion assays. For example, Wu et al suggested that actin dynamics and osmotic pressure are responsible for the omega shape shrinking, whereas actin and calcium-dependent dynamics regulated the size of the fusion pore, both regulatory elements not found in most artificial systems reconstituting fusion (Shin et al., 2020). Indeed, the proximity of the cortical actin network to the plasma membrane suggested that actin may also help organize the clustering of the fusion machinery by linking vesicles to the plasma membrane. For example, the Arp2/3 actin nucleator interaction with lipids and signaling proteins, such as PtdIns(4,5)P2 and Cdc42/N-WASP, may link vesicles to the plasma membrane (Figure 3Biii; Gasman et al., 2004). Cortical F-actin was found to control the localization and dynamics of SNAP-25 membrane clusters in neuroendocrine cells (Torregrosa-Hetland et al., 2013). Quantitative imaging of SNAP-25 and the actin probe lifeact-GFP revealed colocalization at the plasma membrane, indicating the association of secretory machinery to the F-actin cortex. In agreement with these data, Yuan et al. (2015) found evidence that the organization of fusion hotspots in insulin-secreting INS-1 cells relied on the cytoskeleton. Using TIRF microscopy they found that individual fusion events were clustered and that this clustering disappeared upon inhibition of either the actin cytoskeleton using cytochalasin D or microtubule cytoskeleton with nocodazole. Defining how the cytoskeleton regulates fusion and the plasma membrane remains a complicated area of study, due to the pleiotropic effects of manipulating any single cytoskeletal element.

In addition to the actin cytoskeleton, several other proteins such as dynamin, alpha-synuclein, complexin, synaptotagmin, and TRIM67 regulate fusion pore kinetics and fusion mode (Figure 3Biv). Both dynamin and alpha-synuclein alter the kinetics of fusion pore dilation and the mode of exocytosis. Dynamin I is enriched in neurons, while dynamin I and II are found in neuroendocrine cells, where they regulate the amount of exocytic cargo release (González-Jamett et al., 2013; Jackson et al., 2015). Anantharam et al. (2011) found that when dynamin I GTPase activity was reduced, a slower onset of pore dilation occurred along with increased FVF (Anantharam et al., 2011; Jackson et al., 2015). Neuroendocrine cells transfected with a dynamin I mutant with reduced GTPase activity compared to wildtype dynamin I showed an increase in the length of the pre-spike foot of amperometry measurements of catecholamine, suggesting a slower onset of pore dilation. Treatment of neuroendocrine cells with the dynamin activator Ryngo, however, slowed the kinetics of FVF events and decreased the number of KNR events (Jackson et al., 2015). This suggested that dynamin I was a tuneable regulator of both kinetics and mode of exocytosis. Overexpression of alpha-synuclein, a protein that localizes to the presynapse, altered fusion pore kinetics, reducing the time until the full release of BDNF-pHluorin from vesicles (Logan et al., 2017). Unlike dynamin I overexpression, however, the faster kinetics of fusion pore dilation was not accompanied by decreased FVF, but an increased number of presumed FVF events. This suggested that modes of fusion may be regulated distinctly from fusion pore kinetics. Recently we described the brain enriched E3 ubiquitin ligase TRIM67 as a regulator of fusion mode in developing cortical neurons (Urbina et al., 2020). Deletion of *Trim67* led to a decrease in FVFi and FVFd classified VAMP2-pHluorin events, and an increase in KNRi and KNRd events, with no change in the total frequency of exocytic events. Further investigation suggested that TRIM67 acted to decrease levels of SNAP-47 and reduced the level of SNAP-47 in complex with other SNAREs. Deletion of *Trim67, therefore*, caused an increase in SNAP-47 levels, altered SNARE complex composition, and altered fusion behavior that suggested SNAP-47 containing SNARE complexes may have reduced ability to dilate the fusion pore after opening. Knocking down SNAP-47 with siRNA reduced KNRi and KNRd in *Trim67*^−/−^ neurons. These results suggested that altering SNARE complex composition as a way to regulate the mode of fusion.

While dynamin and alpha-synuclein altered fusion pore dilation rate and mode of exocytosis, other proteins, such as complexin II, are suggested to bind SNARE complexes and stabilize the fusion pore (Archer et al., 2002). Overexpression of complexin II in neuroendocrine cells reduced the number of exocytic events and decreased amperometrically measured fusion rate, rise time, and fall time of fusion events, consistent with induced earlier fusion pore closure and kiss-and-run exocytosis. Unlike dynamin or alpha-synuclein, however, complexin II did not modify the rate of fusion pore expansion but instead limited the time over which cargo release occurs. Another mechanism for fusion pore regulation briefly touched upon here suggests that distinct populations of vesicles with different cargo exist, which may also have different fusion machinery. Synaptotagmins are a family of membrane trafficking proteins, several of which act as calcium sensors in the regulation of neurotransmitter release including synaptotagmin-1 and synaptotagmin-7 (Chapman, 2008). Synaptotagmin-1 regulated fusion exhibited slower fusion kinetics and was less spatially-clustered than synaptotagmin-7 fusion, suggesting diversity in vesicle population fusion (Rao et al., 2017). One question that remains is whether different vesicle populations exist at different time points in development, and whether the mode of fusion changes based on the developmental need of the neuron.

## Conclusion

Although SNARE-mediated fusion is an essential and well-known mechanism that drives the synaptic transmission and neuron development and growth, several questions remain as to the regulation of the fusion pore. A large and rich body of evidence has been gathered since SNAREs were first discovered, suggesting a highly-regulatable and tuneable system of cargo release and membrane insertion. Emerging evidence concerning the existence of new modes of exocytosis, differences in SNARE activity at different developmental stages, and the number of elements that SNARE-mediated fusion regulate continues to expand and drive the field towards the new question and ultimately new insights into SNARE-mediated exocytosis.

## Author Contributions

FU and SG wrote and edited the manuscript. FU made figures and performed sequence analysis.

## Conflict of Interest

The authors declare that the research was conducted in the absence of any commercial or financial relationships that could be construed as a potential conflict of interest.

## References

[B1] Adolfsen B. Saraswati S. Yoshihara M. Troy Littleton J. (2004). Synaptotagmins are trafficked to distinct subcellular domains including the postsynaptic compartment. J. Cell Biol. 166, 249–260. 10.1083/jcb.200312054 15263020 PMC2172321

[B2] Advani R. J. Bae H. R. Bock J. B. Chao D. S. Doung Y. C. Prekeris R.. (1998). Seven novel mammalian SNARE proteins localize to distinct membrane compartments. J. Biol. Chem. 273, 10317–10324. 10.1074/jbc.273.17.10317 9553086

[B1000] Akiyama H. Kamiguchi H. (2015). Second messenger networks for accurate growth cone guidance. Dev. Neurobiol. 75, 411–422. 10.1002/dneu.22157 24285606

[B300] Akiyama H. Kamiguchi H. (2010). Phosphatidylinositol 3-kinase facilitates microtubule-dependent membrane transport for neuronal growth cone guidance. J. Biol. Chem. 285, 41740–41748. 10.1074/jbc.M110.156489 21041312 PMC3009901

[B301] Albillos A. Dernick G. Horstmann H. Almers W. de Toledo G. A. Lindau M. (1997). The exocytotic event in chromaffin cells revealed by patch amperometry. Nature 389, 509–512. 10.1038/39081 9333242

[B3] Anantharam A. Bittner M. A. Aikman R. L. Stuenkel E. L. Schmid S. L. Axelrod D.. (2011). A new role for the dynamin GTPase in the regulation of fusion pore expansion. Mol. Biol. Cell 22, 1907–1918. 10.1091/mbc.e11-02-0101 21460182 PMC3103406

[B4] Aravanis A. M. Pyle J. L. Tsien R. W. (2003). Single synaptic vesicles fusing transiently and successively without loss of identity. Nature 423, 643–647. 10.1038/nature01686 12789339

[B5] Archer D. A. Graham M. E. Burgoyne R. D. (2002). Complexin regulates the closure of the fusion pore during regulated vesicle exocytosis. J. Biol. Chem. 277, 18249–18252. 10.1074/jbc.c200166200 11929859

[B6] Arora S. Saarloos I. Kooistra R. van de Bospoort R. Verhage M. Toonen R. F. (2017). SNAP-25 gene family members differentially support secretory vesicle fusion. J. Cell Sci. 130, 1877–1889. 10.1242/jcs.201889 28404788

[B7] Azevedo F. A. C. Carvalho L. R. B. Grinberg L. T. Farfel J. M. Ferretti R. E. L. Leite R. E. P.. (2009). Equal numbers of neuronal and nonneuronal cells make the human brain an isometrically scaled-up primate brain. J. Comp. Neurol. 513, 532–541. 10.1002/cne.21974 19226510

[B8] Baas P. W. Deitch J. S. Black M. M. Banker G. A. (1988). Polarity orientation of microtubules in hippocampal neurons: uniformity in the axon and nonuniformity in the dendrite. Proc. Natl. Acad. Sci. U S A 85, 8335–8339. 10.1073/pnas.85.21.8335 3054884 PMC282424

[B9] Balkowiec A. Katz D. M. (2002). Cellular mechanisms regulating activity-dependent release of native brain-derived neurotrophic factor from hippocampal neurons. J. Neurosci. 22, 10399–10407. 10.1523/jneurosci.22-23-10399.2002 12451139 PMC6758764

[B10] Bao H. Das D. Courtney N. A. Jiang Y. Briguglio J. S. Lou X.. (2018). Dynamics and number of trans-SNARE complexes determine nascent fusion pore properties. Nature 554, 260–263. 10.1038/nature25481 29420480 PMC5808578

[B302] Bello O. D. Auclair S. M. Rothman J. E. Krishnakumar S. S. (2016). Using ApoE nanolipoprotein particles to analyze SNARE-induced fusion pores. Langmuir 32, 3015–3023. 10.1021/acs.langmuir.6b00245 26972604 PMC4946868

[B11] Bilimoria P. M. Bonni A. (2013). Molecular control of axon branching. Neuroscientist 19, 16–24. 10.1177/1073858411426201 22179123 PMC3490022

[B12] Block M. R. Glick B. S. Wilcox C. A. Wieland F. T. Rothman J. E. (1988). Purification of an N-ethylmaleimide-sensitive protein catalyzing vesicular transport. Proc. Natl. Acad. Sci. 85, 7852–7856. 10.1073/pnas.85.21.7852 3186695 PMC282295

[B13] Bock J. B. Klumperman J. Davanger S. Scheller R. H. (1997). Syntaxin 6 functions in trans-Golgi network vesicle trafficking. Mol. Biol. Cell 8, 1261–1271. 10.1091/mbc.8.7.1261 9243506 PMC276151

[B14] Bock J. B. Matern H. T. Peden A. A. Scheller R. H. (2001). A genomic perspective on membrane compartment organization. Nature 409, 839–841. 10.1038/35057024 11237004

[B15] Bowser D. N. Khakh B. S. (2007). Two forms of single-vesicle astrocyte exocytosis imaged with total internal reflection fluorescence microscopy. Proc. Natl. Acad. Sci. U S A 104, 4212–4217. 10.1073/pnas.0607625104 17360502 PMC1820734

[B16] Bradke F. Dotti C. G. (1997). Neuronal polarity: vectorial cytoplasmic flow precedes axon formation. Neuron 19, 1175–1186. 10.1016/s0896-6273(00)80410-9 9427242

[B303] Bray D. (1970). Surface movements during the growth of single explanted neurons. Proc. Natl. Acad. Sci. U S A 65, 905–910. 10.1073/pnas.65.4.905 5266160 PMC283002

[B17] Bretou M. Jouannot O. Fanget I. Pierobon P. Larochette N. Gestraud P.. (2014). Cdc42 controls the dilation of the exocytotic fusion pore by regulating membrane tension. Mol. Biol. Cell 25, 3195–3209. 10.1091/mbc.e14-07-1229 25143404 PMC4196869

[B304] Burgo A. Proux-Gillardeaux V. Sotirakis E. Bun P. Casano A. Verraes A.. (2012). A molecular network for the transport of the TI-VAMP/VAMP7 vesicles from cell center to periphery. Dev. Cell 23, 166–180. 10.1016/j.devcel.2012.04.019 22705394

[B18] Cardoso-Moreira M. Halbert J. Valloton D. Velten B. Chen C. Shao Y.. (2019). Gene expression across mammalian organ development. Nature 571, 505–509. 10.1038/s41586-019-1338-5 31243369 PMC6658352

[B19] Ceccarelli B. Hurlbut W. P. Mauro A. (1972). Depletion of vesicles from frog neuromuscular junctions by prolonged tetanic stimulation. J. Cell Biol. 54, 30–38. 10.1083/jcb.54.1.30 4338962 PMC2108853

[B20] Chapman E. R. (2008). How does synaptotagmin trigger neurotransmitter release? Annu. Rev. Biochem. 77, 615–641. 10.1146/annurev.biochem.77.062005.101135 18275379

[B21] Cheng J. Liu W. Duffney L. J. Yan Z. (2013). SNARE proteins are essential in the potentiation of NMDA receptors by group II metabotropic glutamate receptors. J. Physiol. 591, 3935–3947. 10.1113/jphysiol.2013.255075 23774277 PMC3764638

[B22] Clary D. O. Griff I. C. Rothman J. E. (1990). SNAPs, a family of NSF attachment proteins involved in intracellular membrane fusion in animals and yeast. Cell 61, 709–721. 10.1016/0092-8674(90)90482-t 2111733

[B23] Cooper J. A. (2013). Mechanisms of cell migration in the nervous system. J. Cell Biol. 202, 725–734. 10.1083/jcb.201305021 23999166 PMC3760606

[B24] Cotrufo T. Andrés R. M. Ros O. Pérez-Brangulí F. Muhaisen A. Fuschini G.. (2012). Syntaxin 1 is required for DCC/Netrin-1-dependent chemoattraction of migrating neurons from the lower rhombic lip. Eur. J. Neurosci. 36, 3152–3164. 10.1111/j.1460-9568.2012.08259.x 22946563

[B25] Craig A. M. Wyborski R. J. Banker G. (1995). Preferential addition of newly synthesized membrane protein at axonal growth cones. Nature 375, 592–594. 10.1038/375592a0 7791876

[B26] Crary J. L. Haldar K. (1992). Brefeldin A inhibits protein secretion and parasite maturation in the ring stage of Plasmodium falciparum. Mol. Biochem. Parasitol. 53, 185–192. 10.1016/0166-6851(92)90020-k 1501638

[B27] Diz-Muñoz A. Fletcher D. A. Weiner O. D. (2013). Use the force: membrane tension as an organizer of cell shape and motility. Trends Cell Biol. 23, 47–53. 10.1016/j.tcb.2012.09.006 23122885 PMC3558607

[B28] Dotti C. G. Sullivan C. A. Banker G. A. (1988). The establishment of polarity by hippocampal neurons in culture. J. Neurosci. 8, 1454–1468. 10.1523/jneurosci.08-04-01454.1988 3282038 PMC6569279

[B29] Drachman D. A. (2005). Do we have brain to spare? Neurology 64, 2004–2005. 10.1212/01.wnl.0000166914.38327.bb 15985565

[B305] Dudanova I. Klein R. (2013). Integration of guidance cues: parallel signaling and crosstalk. Trends Neurosci. 36, 295–304. 10.1016/j.tins.2013.01.007 23485451

[B30] Fasshauer D. Sutton R. B. Brunger A. T. Jahn R. (1998). Conserved structural features of the synaptic fusion complex: SNARE proteins reclassified as Q- and R-SNAREs. Proc. Natl. Acad. Sci. 95, 15781–15786. 10.1073/pnas.95.26.15781 9861047 PMC28121

[B31] Fernandez J. M. Neher E. Gomperts B. D. (1984). Capacitance measurements reveal stepwise fusion events in degranulating mast cells. Nature 312, 453–455. 10.1038/312453a0 6504157

[B32] Frischknecht R. Fejtova A. Viesti M. Stephan A. Sonderegger P. (2008). Activity-induced synaptic capture and exocytosis of the neuronal serine protease neurotrypsin. J. Neurosci. 28, 1568–1579. 10.1523/jneurosci.3398-07.2008 18272678 PMC6671550

[B33] Frühbeis C. Fröhlich D. Kuo W. P. Amphornrat J. Thilemann S. Saab A. S.. (2013). Neurotransmitter-triggered transfer of exosomes mediates oligodendrocyte-neuron communication. PLoS Biol. 11:e1001604. 10.1371/journal.pbio.1001604 23874151 PMC3706306

[B34] Gandhi S. P. Stevens C. F. (2003). Three modes of synaptic vesicular recycling revealed by single-vesicle imaging. Nature 423, 607–613. 10.1038/nature01677 12789331

[B35] Gao Y. Zorman S. Gundersen G. Xi Z. Ma L. Sirinakis G.. (2012). Single reconstituted neuronal SNARE complexes zipper in three distinct stages. Science 337, 1340–1343. 10.1126/science.1224492 22903523 PMC3677750

[B307] Gasman S. Chasserot-Golaz S. Malacombe M. Way M. Bader M.-F. (2004). Regulated exocytosis in neuroendocrine cells: a role for subplasmalemmal Cdc42/N-WASP-induced actin filaments. Mol. Biol. Cell 15, 520–531. 10.1091/mbc.e03-06-0402 14617808 PMC329227

[B36] Gauthier N. C. Fardin M. A. Roca-Cusachs P. Sheetz M. P. (2011). Temporary increase in plasma membrane tension coordinates the activation of exocytosis and contraction during cell spreading. Proc. Natl. Acad. Sci. U S A 108, 14467–14472. 10.1073/pnas.1105845108 21808040 PMC3167546

[B308] Gomez T. M. Zheng J. Q. (2006). The molecular basis for calcium-dependent axon pathfinding. Nat. Rev. Neurosci. 7, 115–125. 10.1038/nrn1844 16429121

[B37] González-Jamett A. M. Momboisse F. Guerra M. J. Ory S. Báez-Matus X. Barraza N.. (2013). Dynamin-2 regulates fusion pore expansion and quantal release through a mechanism that involves actin dynamics in neuroendocrine chromaffin cells. PLoS One 8:e70638. 10.1371/journal.pone.0070638 23940613 PMC3734226

[B38] Grafmüller A. Shillcock J. Lipowsky R. (2009). The fusion of membranes and vesicles: pathway and energy barriers from dissipative particle dynamics. Biophys. J. 96, 2658–2675. 10.1016/j.bpj.2008.11.073 19348749 PMC2711276

[B309] Grassi D. Plonka F. B. Oksdath M. Guil A. N. Sosa L. J. Quiroga S. (2015). Selected SNARE proteins are essential for the polarized membrane insertion of igf-1 receptor and the regulation of initial axonal outgrowth in neurons. Cell Discov. 1:15023. 10.1038/celldisc.2015.23 27462422 PMC4860833

[B39] Gupton S. L. Gertler F. B. (2010). Integrin signaling switches the cytoskeletal and exocytic machinery that drives neuritogenesis. Dev. Cell 18, 725–736. 10.1016/j.devcel.2010.02.017 20493807 PMC3383070

[B40] Harata N. C. Choi S. Pyle J. L. Aravanis A. M. Tsien R. W. (2006). Frequency-dependent kinetics and prevalence of kiss-and-run and reuse at hippocampal synapses studied with novel quenching methods. Neuron 49, 243–256. 10.1016/j.neuron.2005.12.018 16423698

[B41] Hartmann M. Heumann R. Lessmann V. (2001). Synaptic secretion of BDNF after high-frequency stimulation of glutamatergic synapses. EMBO J. 20, 5887–5897. 10.1093/emboj/20.21.5887 11689429 PMC125691

[B42] Heuser J. E. (1989). Review of electron microscopic evidence favouring vesicle exocytosis as the structural basis for quantal release during synaptic transmission. Q. J. Exp. Physiol. 74, 1051–1069. 10.1113/expphysiol.1989.sp003333 2560556

[B43] Heuser J. E. Reese T. S. (1973). Evidence for recycling of synaptic vesicle membrane during transmitter release at the frog neuromuscular junction. J. Cell Biol. 57, 315–344. 10.1083/jcb.57.2.315 4348786 PMC2108984

[B44] Hirling H. Steiner P. Chaperon C. Marsault R. Regazzi R. Catsicas S. (2000). Syntaxin 13 is a developmentally regulated SNARE involved in neurite outgrowth and endosomal trafficking. Eur. J. Neurosci. 12, 1913–1923. 10.1046/j.1460-9568.2000.00076.x 10886332

[B45] Holt M. Varoqueaux F. Wiederhold K. Takamori S. Urlaub H. Fasshauer D.. (2006). Identification of SNAP-47, a novel Qbc-SNARE with ubiquitous expression. J. Biol. Chem. 281, 17076–17083. 10.3410/f.1033038.375051 16621800

[B310] Horton A. C. Rácz B. Monson E. E. Lin A. L. Weinberg R. J. Ehlers M. D. (2005). Polarized secretory trafficking directs cargo for asymmetric dendrite growth and morphogenesis. Neuron 48, 757–771. 10.1016/j.neuron.2005.11.005 16337914

[B46] Hu C. Ahmed M. Melia T. J. Söllner T. H. Mayer T. Rothman J. E. (2003). Fusion of cells by flipped SNAREs. Science 300, 1745–1749. 10.1126/science.1084909 12805548

[B47] Hunt D. L. Castillo P. E. (2012). Synaptic plasticity of NMDA receptors: mechanisms and functional implications. Curr. Opin. Neurobiol. 22, 496–508. 10.1016/j.conb.2012.01.007 22325859 PMC3482462

[B2000] Igarashi M. Kozaki S. Terakawa S. Kawano S. Ide C. Komiya Y. (1996). Growth cone collapse and inhibition of neurite growth by Botulinum neurotoxin C1: a t-SNARE is involved in axonal growth. J. Cell Biol. 134, 205–215. 10.1083/jcb.134.1.205 8698815 PMC2120926

[B311] Igarashi M. Tagaya M. Komiya Y. (1997). The soluble N-ethylmaleimide-sensitive factor attached protein receptor complex in growth cones: molecular aspects of the axon terminal development. J. Neurosci. 17, 1460–1470. 10.1523/jneurosci.17-04-01460.1997 9006987 PMC6793737

[B48] Jackson J. Papadopulos A. Meunier F. A. McCluskey A. Robinson P. J. Keating D. J. (2015). Small molecules demonstrate the role of dynamin as a bi-directional regulator of the exocytosis fusion pore and vesicle release. Mol. Psychiatry 20, 810–819. 10.1038/mp.2015.56 25939402

[B49] Jahn R. Fasshauer D. (2012). Molecular machines governing exocytosis of synaptic vesicles. Nature 490, 201–207. 10.1038/nature11320 23060190 PMC4461657

[B50] Jung J.-J. Inamdar S. M. Tiwari A. Choudhury A. (2012). Regulation of intracellular membrane trafficking and cell dynamics by syntaxin-6. Biosci. Rep. 32, 383–391. 10.1042/bsr20120006 22489884 PMC3392101

[B312] Kabayama H. Tokushige N. Takeuchi M. Mikoshiba K. (2008). Syntaxin 6 regulates nerve growth factor-dependent neurite outgrowth. Neurosci. Lett. 436, 340–344. 10.1016/j.neulet.2008.03.061 18406529

[B51] Kaibuchi K. (2009). Neuronal polarity: from extracellular signals to intracellular mechanisms. Neurosci. Res. 65:S2. 10.1016/j.neures.2009.09.013 17311006

[B52] Kloepper T. H. Nickias Kienle C. Fasshauer D. (2007). An elaborate classification of SNARE proteins sheds light on the conservation of the eukaryotic endomembrane system. Mol. Biol. Cell 18, 3463–3471. 10.1091/mbc.e07-03-0193 17596510 PMC1951749

[B53] Kusumi A. Nakada C. Ritchie K. Murase K. Suzuki K. Murakoshi H.. (2005). Paradigm shift of the plasma membrane concept from the two-dimensional continuum fluid to the partitioned fluid: high-speed single-molecule tracking of membrane molecules. Annu. Rev. Biophys. Biomol. Struct. 34, 351–378. 10.1146/annurev.biophys.34.040204.144637 15869394

[B54] La Manno G. Gyllborg D. Codeluppi S. Nishimura K. Salto C. Zeisel A.. (2016). Molecular diversity of midbrain development in mouse, human and stem cells. Cell 167, 566.e19–580.e19. 10.1016/j.cell.2016.09.02 27716510 PMC5055122

[B55] Lau C. G. Takayasu Y. Rodenas-Ruano A. Paternain A. V. Lerma J. Bennett M. V. L.. (2010). SNAP-25 is a target of protein kinase C phosphorylation critical to NMDA receptor trafficking. J. Neurosci. 30, 242–254. 10.1523/jneurosci.4933-08.2010 20053906 PMC3397691

[B56] Le N. Q. K. Huynh T.-T. (2019). Identifying SNAREs by incorporating deep learning architecture and amino acid embedding representation. Front. Physiol. 10:1501. 10.3389/fphys.2019.01501 31920706 PMC6914855

[B57] Le N. Q. K. Nguyen V.-N. (2019). SNARE-CNN: a 2D convolutional neural network architecture to identify SNARE proteins from high-throughput sequencing data. PeerJ Comput. Sci. 5:e177 10.7287/peerj-cs.177v0.1/reviews/1 PMC7924420 33816830

[B58] Lewis S. (2013). Transporting cargo from A to B. Nat. Rev. Neurosci. 14, 589–589. 10.1038/nrn3568 23900413

[B59] Liu W. Montana V. Bai J. Chapman E. R. Mohideen U. Parpura V. (2006). Single molecule mechanical probing of the SNARE protein interactions. Biophys. J. 91, 744–758. 10.1529/biophysj.105.073312 16648158 PMC1483094

[B60] Logan T. Bendor J. Toupin C. Thorn K. Edwards R. H. (2017). α-synuclein promotes dilation of the exocytotic fusion pore. Nat‥ Neurosci. 20, 681–689. 10.1038/nn.4529 28288128 PMC5404982

[B313] Lu X. Zhang F. McNew J. A. Shin Y.-K. (2005). Membrane fusion induced by neuronal SNAREs transits through hemifusion. J. Biol. Chem. 280, 30538–30541. 10.1074/jbc.m506862200 15980065

[B61] Lundberg J. M. Rudehill A. Sollevi A. Theodorsson-Norheim E. Hamberger B. (1986). Frequency- and reserpine-dependent chemical coding of sympathetic transmission: differential release of noradrenaline and neuropeptide Y from pig spleen. Neurosci. Lett. 63, 96–100. 10.1016/0304-3940(86)90020-0 3005926

[B62] Lüscher C. Isaac J. T. (2009). The synapse: center stage for many brain diseases. J. Physiol. 587, 727–729. 10.1113/jphysiol.2008.167742 19074963 PMC2669966

[B63] Malhotra V. (1988). Role of an N-ethylmaleimide-sensitive transport component in promoting fusion of transport vesicles with cisternae of the Golgi stack. Cell 54, 221–227. 10.1016/0092-8674(88)90554-5 3390865

[B314] Martinez-Arca S. Coco S. Mainguy G. Schenk U. Alberts P. Bouillé P.. (2001). A common exocytotic mechanism mediates axonal and dendritic outgrowth. J. Neurosci. 21, 3830–3838. 10.1523/JNEUROSCI.21-11-03830.2001 11356871 PMC6762683

[B3000] McDargh Z. A. Polley A. O’Shaughnessy B. (2018). SNARE-mediated membrane fusion is a two-stage process driven by entropic forces. FEBS Lett. 592, 3504–3515. 10.1002/1873-3468.13277 30346036 PMC6298751

[B64] Merighi A. (2018). Costorage of high molecular weight neurotransmitters in large dense core vesicles of mammalian neurons. Front. Cell. Neurosci. 12:272. 10.3389/fncel.2018.00272 30186121 PMC6110924

[B65] Micheva K. D. Busse B. Weiler N. C. O’Rourke N. Smith S. J. (2010). Single-synapse analysis of a diverse synapse population: proteomic imaging methods and markers. Neuron 68, 639–653. 10.1016/j.neuron.2010.09.024 21092855 PMC2995697

[B66] Miesenböck G. De Angelis D. A. Rothman J. E. (1998). Visualizing secretion and synaptic transmission with pH-sensitive green fluorescent proteins. Nature 394, 192–195. 10.1038/28190 9671304

[B67] Min D. Kim K. Hyeon C. Cho Y. H. Shin Y.-K. Yoon T.-Y. (2013). Mechanical unzipping and rezipping of a single SNARE complex reveals hysteresis as a force-generating mechanism. Nat. Commun. 4:1705. 10.1038/ncomms2692 23591872 PMC3644077

[B68] Molnár Z. López-Bendito G. Small J. Partridge L. D. Blakemore C. Wilson M. C. (2002). Normal development of embryonic thalamocortical connectivity in the absence of evoked synaptic activity. J. Neurosci. 22, 10313–10323. 10.1523/jneurosci.22-23-10313.2002 12451131 PMC6758728

[B315] Mostafavi H. Thiyagarajan S. Stratton B. S. Karatekin E. Warner J. M. Rothman J. E.. (2017). Entropic forces drive self-organization and membrane fusion by SNARE proteins. Proc. Natl. Acad. Sci. U S A 114, 5455–5460. 10.1073/pnas.1611506114 28490503 PMC5448213

[B69] Mouritsen O. G. Bloom M. (1984). Mattress model of lipid-protein interactions in membranes. Biophys. J. 46, 141–153. 10.1016/s0006-3495(84)84007-2 6478029 PMC1435039

[B70] Mullock B. M. Smith C. W. Ihrke G. Bright N. A. Lindsay M. Parkinson E. J.. (2000). Syntaxin 7 is localized to late endosome compartments, associates with Vamp 8 and Is required for late endosome-lysosome fusion. Mol. Biol. Cell 11, 3137–3153. 10.1091/mbc.11.9.3137 10982406 PMC14981

[B71] Nath V. R. Mishra S. Basak B. Trivedi D. Raghu P. (2019). Extended synaptotagmin regulates plasma membrane-endoplasmic reticulum contact site structure and lipid transfer function *in vivo*. bioRxiv [Preprint]. 10.1101/2019.12.12.874933 PMC7507014 32716137

[B72] Novick P. Schekman R. (1979). Secretion and cell-surface growth are blocked in a temperature-sensitive mutant of Saccharomyces cerevisiae. Proc. Natl. Acad. Sci. 76, 1858–1862. 10.1073/pnas.76.4.1858 377286 PMC383491

[B73] Osen-Sand A. Staple J. K. Naldi E. Schiavo G. Rossetto O. Petitpierre S.. (1996). Common and distinct fusion proteins in axonal growth and transmitter release. J. Comp. Neurol. 367, 222–234. 8708006 10.1002/(SICI)1096-9861(19960401)367:2<222::AID-CNE5>3.0.CO;2-7

[B74] Parlati F. McNew J. A. Fukuda R. Miller R. Söllner T. H. Rothman J. E. (2000). Topological restriction of SNARE-dependent membrane fusion. Nature 407, 194–198. 10.1038/35025076 11001058

[B75] Parlati F. Varlamov O. Paz K. McNew J. A. Hurtado D. Söllner T. H.. (2002). Distinct SNARE complexes mediating membrane fusion in Golgi transport based on combinatorial specificity. Proc. Natl. Acad. Sci. U S A 99, 5424–5429. 10.1073/pnas.082100899 11959998 PMC122785

[B76] Pfenninger K. H. (2009). Plasma membrane expansion: a neuron’s Herculean task. Nat. Rev. Neurosci. 10, 251–261. 10.1038/nrn2593 19259102

[B77] Pfenninger K. H. Friedman L. B. (1993). Sites of plasmalemmal expansion in growth cones. Brain Res. Dev. Brain Res. 71, 181–192. 10.1016/0165-3806(93)90170-f 8491040

[B78] Popov S. Brown A. Poo M. (1993). Forward plasma membrane flow in growing nerve processes. Science 259, 244–246. 10.1126/science.7678471 7678471

[B316] Prekeris R. Yang B. Oorschot V. Klumperman J. Scheller R. H. (1999). Differential roles of syntaxin 7 and syntaxin 8 in endosomal trafficking. Mol. Biol. Cell 10, 3891–3908. 10.1091/mbc.10.11.3891 10564279 PMC25687

[B79] Qu L. Akbergenova Y. Hu Y. Schikorski T. (2009). Synapse-to-synapse variation in mean synaptic vesicle size and its relationship with synaptic morphology and function. J. Comp. Neurol. 514, 343–352. 10.1002/cne.22007 19330815

[B80] Rao T. C. Rodriguez Z. S. Bradberry M. M. Ranski A. H. Dahl P. J. Schmidtke M. W.. (2017). Synaptotagmin isoforms confer distinct activation kinetics and dynamics to chromaffin cell granules. J. Gen. Physiol. 149, 763–780. 10.1085/jgp.201711757 28687607 PMC5560776

[B81] Richards D. A. (2009). Vesicular release mode shapes the postsynaptic response at hippocampal synapses. J. Physiol. 587, 5073–5080. 10.1113/jphysiol.2009.175315 19752123 PMC2790249

[B317] Risselada H. J. Smirnova Y. Grubmüller H. (2014). Free energy landscape of rim-pore expansion in membrane fusion. Biophys. J. 107, 2287–2295. 10.1016/j.bpj.2014.08.022 25418297 PMC4241460

[B82] Rizzoli S. O. Jahn R. (2007). Kiss-and-run, collapse and “readily retrievable” vesicles. Traffic 8, 1137–1144. 10.1111/j.1600-0854.2007.00614.x 17645434

[B318] Ros O. Cotrufo T. Martínez-Mármol R. Soriano E. (2015). Regulation of patterned dynamics of local exocytosis in growth cones by netrin-1. J. Neurosci. 35, 5156–5170. 10.1523/JNEUROSCI.0124-14.2015 25834042 PMC6705414

[B83] Rothman J. E. Orci L. (1992). Molecular dissection of the secretory pathway. Nature 355, 409–415. 10.1038/355409a0 1734280

[B319] Sabo S. L. McAllister A. K. (2003). Mobility and cycling of synaptic protein-containing vesicles in axonal growth cone filopodia. Nat. Neurosci. 6, 1264–1269. 10.1038/nn1149 14608359

[B84] Sampo B. Kaech S. Kunz S. Banker G. (2003). Two distinct mechanisms target membrane proteins to the axonal surface. Neuron 37, 611–624. 10.1016/s0896-6273(03)00058-8 12597859

[B85] Schoch S. Deák F. Königstorfer A. Mozhayeva M. Sara Y. Südhof T. C.. (2001). SNARE function analyzed in synaptobrevin/VAMP knockout mice. Science 294, 1117–1122. 10.1126/science.1064335 11691998

[B86] Schulz J. B. Hausmann L. (2016). Synaptopathies: synaptic dysfunction in neurological disorders—a review written by students for students and a story of success for ISN schools. J. Neurochem. 138, 783–784. 10.1111/jnc.13755 27604179

[B87] Shah N. Klausner R. D. (1993). Brefeldin A reversibly inhibits secretion in Saccharomyces cerevisiae. J. Biol. Chem. 268, 5345–5348. 8449896

[B320] Shi L. Shen Q.-T. Kiel A. Wang J. Wang H.-W. Melia T. J.. (2012). SNARE proteins: one to fuse and three to keep the nascent fusion pore open. Science 335, 1355–1359. 10.1126/science.1214984 22422984 PMC3736847

[B88] Shi Z. Graber Z. T. Baumgart T. Stone H. A. Cohen A. E. (2018). Cell membranes resist flow. Cell 175, 1769.e13–1779.e13. 10.1016/j.cell.2018.09.054 30392960 PMC6541487

[B89] Shimojo M. Courchet J. Pieraut S. Torabi-Rander N. Sando R. 3rd Polleux F.. (2015). SNAREs controlling vesicular release of BDNF and development of callosal axons. Cell Rep. 11, 1054–1066. 10.1016/j.celrep.2015.04.032 25959820 PMC4439258

[B90] Shin W. Arpino G. Thiyagarajan S. Su R. Ge L. McDargh Z.. (2020). Vesicle shrinking and enlargement play opposing roles in the release of exocytotic contents. Cell Rep. 30, 421.e7–431.e7. 10.1016/j.celrep.2019.12.044 31940486 PMC7010319

[B91] Shin W. Ge L. Arpino G. Villarreal S. A. Hamid E. Liu H.. (2018). Visualization of membrane pore in live cells reveals a dynamic-pore theory governing fusion and endocytosis. Cell 173, 934.e12–945.e12. 10.1016/j.cell.2018.02.062 29606354 PMC5935532

[B92] Söllner T. Whiteheart S. W. Brunner M. Erdjument-Bromage H. Geromanos S. Tempst P.. (1993). SNAP receptors implicated in vesicle targeting and fusion. Nature 362, 318–324. 10.1038/362318a0 8455717

[B93] Sørensen J. B. Nagy G. Varoqueaux F. Nehring R. B. Brose N. Wilson M. C.. (2003). Differential control of the releasable vesicle pools by SNAP-25 splice variants and SNAP-23. Cell 114, 75–86. 10.1016/s0092-8674(03)00477-x 12859899

[B94] Sosa L. Dupraz S. Laurino L. Bollati F. Bisbal M. Cáceres A.. (2006). IGF-1 receptor is essential for the establishment of hippocampal neuronal polarity. Nat. Neurosci. 9, 993–995. 10.1038/nn1742 16845384

[B95] Sperotto M. M. Ipsen J. H. Mouritsen O. G. (1989). Theory of protein-induced lateral phase separation in lipid membranes. Cell Biophys. 14, 79–95. 10.1007/bf02797393 2465088

[B96] Staal R. G. W. Mosharov E. V. Sulzer D. (2004). Dopamine neurons release transmitter *via* a flickering fusion pore. Nat. Neurosci. 7, 341–346. 10.1038/nn1205 14990933

[B97] Steegmaier M. Klumperman J. Foletti D. L. Yoo J.-S. Scheller R. H. (1999). Vesicle-associated membrane protein 4 is implicated in trans-golgi network vesicle trafficking. Mol. Biol. Cell 10, 1957–1972. 10.1091/mbc.10.6.1957 10359608 PMC25394

[B98] Steegmaier M. Yang B. Yoo J. S. Huang B. Shen M. Yu S.. (1998). Three novel proteins of the syntaxin/SNAP-25 family. J. Biol. Chem. 273, 34171–34179. 10.1074/jbc.273.51.34171 9852078

[B99] Steinman R. M. Mellman I. S. Muller W. A. Cohn Z. A. (1983). Endocytosis and the recycling of plasma membrane. J. Cell Biol. 96, 1–27. 10.1083/jcb.96.1.1 6298247 PMC2112240

[B100] Stepanova T. Slemmer J. Hoogenraad C. C. Lansbergen G. Dortland B. De Zeeuw C. I.. (2003). Visualization of microtubule growth in cultured neurons *via* the use of EB3-GFP (end-binding protein 3-green fluorescent protein). J. Neurosci. 23, 2655–2664. 10.1523/JNEUROSCI.23-07-02655.2003 12684451 PMC6742099

[B101] Stevens C. F. Williams J. H. (2000). “Kiss and run” exocytosis at hippocampal synapses. Proc. Natl. Acad. Sci. 97, 12828–12833. 10.1073/pnas.230438697 11050187 PMC18849

[B321] Stratton B. S. Warner J. M. Wu Z. Nikolaus J. Wei G. Wagnon E.. (2016). Cholesterol increases the openness of SNARE-mediated flickering fusion pores. Biophys. J. 110, 1538–1550. 10.1016/j.bpj.2016.02.019 27074679 PMC4833774

[B102] Südhof T. C. (2004). The synaptic vesicle cycle. Annu. Rev. Neurosci. 27, 509–547. 10.1146/annurev.neuro.26.041002.131412 15217342

[B103] Südhof T. C. Rothman J. E. (2009). Membrane fusion: grappling with SNARE and SM proteins. Science 323, 474–477. 10.1126/science.1161748 19164740 PMC3736821

[B4000] Sutherland D. J. Pujic Z. Goodhill G. J. (2014). Calcium signaling in axon guidance. Trends Neurosci. 37, 424–432. 10.1016/j.tins.2014.05.008 24969461

[B104] Sutton R. B. Bryan Sutton R. Fasshauer D. Jahn R. Brunger A. T. (1998). Crystal structure of a SNARE complex involved in synaptic exocytosis at 2.4 Å resolution. Nature 395, 347–353. 10.1038/26412 9759724

[B322] Tojima T. (2012). Intracellular signaling and membrane trafficking control bidirectional growth cone guidance. Neurosci. Res. 73, 269–274. 10.1016/j.neures.2012.05.010 22684022

[B105] Tojima T. Akiyama H. Itofusa R. Li Y. Katayama H. Miyawaki A.. (2007). Attractive axon guidance involves asymmetric membrane transport and exocytosis in the growth cone. Nat. Neurosci. 10, 58–66. 10.1038/nn1814 17159991

[B323] Tojima T. Hines J. H. Henley J. R. Kamiguchi H. (2011). Second messengers and membrane trafficking direct and organize growth cone steering. Nat. Rev. Neurosci. 12, 191–203. 10.1038/nrn2996 21386859 PMC3133775

[B106] Tojima T. Kamiguchi H. (2015). Exocytic and endocytic membrane trafficking in axon development. Dev. Growth Diff. 57, 291–304. 10.1111/dgd.12218 25966925

[B324] Torregrosa-Hetland C. J. Villanueva J. Garcia-Martínez V. Expósito-Romero G. Francés M. D. M. Gutiérrez L. M. (2013). Cortical F-actin affects the localization and dynamics of SNAP-25 membrane clusters in chromaffin cells. Int. J. Biochem. Cell Biol. 45, 583–592. 10.1016/j.biocel.2012.11.021 23220175

[B107] Urbina F. L. Gomez S. M. Gupton S. L. (2018). Spatiotemporal organization of exocytosis emerges during neuronal shape change. J. Cell Biol. 217, 1113–1128. 10.1083/jcb.201709064 29351997 PMC5839795

[B108] Urbina F. L. Menon S. Goldfarb D. Edwards R. Major M. B. Brennwald P. (2020). TRIM67 regulates exocytic mode and neuronal morphogenesis via SNAP47. bioRxiv [Preprint]. 10.1101/2020.02.01.930404 PMC7941186 33567284

[B109] Washbourne P. Thompson P. M. Carta M. Costa E. T. Mathews J. R. Lopez-Benditó G.. (2002). Genetic ablation of the t-SNARE SNAP-25 distinguishes mechanisms of neuroexocytosis. Nat. Neurosci. 5, 19–26. 10.1038/nn783 11753414

[B110] Weber T. Zemelman B. V. McNew J. A. Westermann B. Gmachl M. Parlati F.. (1998). SNAREpins: minimal machinery for membrane fusion. Cell 92, 759–772. 10.1016/s0092-8674(00)81404-x 9529252

[B111] Weimbs T. Low S. H. Chapin S. J. Mostov K. E. Bucher P. Hofmann K. (1997). A conserved domain is present in different families of vesicular fusion proteins: a new superfamily. Proc. Natl. Acad. Sci. U S A 94, 3046–3051. 10.1073/pnas.94.7.3046 9096343 PMC20319

[B112] Wen P. J. Grenklo S. Arpino G. Tan X. Liao H.-S. Heureaux J.. (2016). Actin dynamics provides membrane tension to merge fusing vesicles into the plasma membrane. Nat. Commun. 7:12604. 10.1038/ncomms12604 27576662 PMC5013665

[B113] Wesolowski J. Caldwell V. Paumet F. (2012). A novel function for SNAP29 (Synaptosomal-Associated Protein of 29 kDa) in mast cell phagocytosis. PLoS One 7:e49886. 10.1371/journal.pone.0049886 23185475 PMC3503860

[B325] Wightman R. M. Haynes C. L. (2004). Synaptic vesicles really do kiss and run. Nat. Neurosci. 7, 321–322. 10.1038/nn0404-321 15048116

[B114] Winkle C. C. McClain L. M. Valtschanoff J. G. Park C. S. Maglione C. Gupton S. L. (2014). A novel Netrin-1-sensitive mechanism promotes local SNARE-mediated exocytosis during axon branching. J. Cell Biol. 205, 217–232. 10.1083/jcb.201311003 24778312 PMC4003241

[B115] Wisco D. Anderson E. D. Chang M. C. Norden C. Boiko T. Fölsch H.. (2003). Uncovering multiple axonal targeting pathways in hippocampal neurons. J. Cell Biol. 162, 1317–1328. 10.1083/jcb.200307069 14517209 PMC2173963

[B116] Wong S. H. Zhang T. Xu Y. Subramaniam V. N. Griffiths G. Hong W. (1998). Endobrevin, a novel synaptobrevin/VAMP-like protein preferentially associated with the early endosome. Mol. Biol. Cell 9, 1549–1563. 10.1091/mbc.9.6.1549 9614193 PMC25382

[B117] Wu Z. Auclair S. M. Bello O. Vennekate W. Dudzinski N. R. Krishnakumar S. S.. (2016). Nanodisc-cell fusion: control of fusion pore nucleation and lifetimes by SNARE protein transmembrane domains. Sci. Rep. 6:27287. 10.1038/srep27287 27264104 PMC4893671

[B118] Wu Z. Bello O. D. Thiyagarajan S. Auclair S. M. Vennekate W. Krishnakumar S. S.. (2017). Dilation of fusion pores by crowding of SNARE proteins. eLife 6:e22964. 10.7554/eLife.22964 28346138 PMC5404929

[B326] Yang L. Dun A. R. Martin K. J. Qiu Z. Dunn A. Lord G. J.. (2012). Secretory vesicles are preferentially targeted to areas of low molecular SNARE density. PLoS One 7:e49514. 10.1371/journal.pone.0049514 23166692 PMC3499460

[B119] Yu H. Liu Y. Gulbranson D. R. Paine A. Rathore S. S. Shen J. (2016). Extended synaptotagmins are Ca2-dependent lipid transfer proteins at membrane contact sites. Proc. Natl. Acad. Sci. 113, 4362–4367. 10.1073/pnas.1517259113 27044075 PMC4843466

[B120] Yuan T. Lu J. Zhang J. Zhang Y. Chen L. (2015). Spatiotemporal detection and analysis of exocytosis reveal fusion “hotspots” organized by the cytoskeleton in endocrine cells. Biophys. J. 108, 251–260. 10.1016/j.bpj.2014.11.3462 25606674 PMC4302205

[B121] Zheng J. Lamoureux P. Santiago V. Dennerll T. Buxbaum R. E. Heidemann S. R. (1991). Tensile regulation of axonal elongation and initiation. J. Neurosci. 11, 1117–1125. 10.1523/jneurosci.11-04-01117.1991 2010807 PMC6575379

[B5000] Zhou Q. Xiao J. Liu Y. (2000). Participation of syntaxin 1A in membrane trafficking involving neurite elongation and membrane expansion. J. Neurosci. Res. 61, 321–328. 10.1002/1097-4547(20000801)61:3<321::aid-jnr10>3.0.co;2-l 10900079

[B122] Zylbersztejn K. Petkovic M. Burgo A. Deck M. Garel S. Marcos S.. (2012). The vesicular SNARE Synaptobrevin is required for Semaphorin 3A axonal repulsion. J. Cell Biol. 196, 37–46. 10.1083/jcb.201106113 22213797 PMC3255983

